# Biomaterials for Modulating the Immune Microenvironment in Rheumatoid Arthritis

**DOI:** 10.34133/bmef.0102

**Published:** 2025-03-10

**Authors:** Qiaoxuan Wang, Junzhang Ji, Ding Huang, Changyou Gao

**Affiliations:** ^1^ MOE Key Laboratory of Macromolecular Synthesis and Functionalization, Department of Polymer Science and Engineering, Zhejiang University, Hangzhou 310058, China.; ^2^ Center for Healthcare Materials, Shaoxing Institute, Zhejiang University, Shaoxing 312099, China.

## Abstract

Rheumatoid arthritis (RA) is a systemic inflammatory autoimmune disease characterized by joint swelling and bone destruction. Despite an incomplete understanding of its genesis, RA is tightly linked to the intricate immunological milieu, involving disruptions in molecular signaling and an imbalance between the innate and adaptive immune systems. With advancements in biomaterials science, the role of biomaterials in RA treatment has evolved from mere drug delivery systems to therapeutic microenvironment modulators, providing drug-independent treatment strategies for RA. In this review, we will delve into the immune microenvironment of RA, focusing on contributions of adaptive immunity, innate immunity, damage-associated molecular patterns (DAMPs), cytokines, and signaling pathways to disease’s pathogenesis and inflammation. We provide a detailed analysis of the applications of novel nonpharmaceutical biomaterials in RA treatment, categorized into 3 key mechanisms: biofactor and signaling pathway regulation, endogenous gas adjustment, and immune cell modulation. The composition, form, therapeutic principles, and treatment efficacy of these biomaterials will be explored. The thorough discussion of these topics will offer a fresh viewpoint on RA treatment strategies and guide future research directions.

## Introduction

Since its identification in 1859 [1], rheumatoid arthritis (RA) has been recognized as a chronic inflammatory autoimmune disorder, primarily affecting the small joints of the hands and feet. This leads to polyarticular, symmetrical, and often aggressive joint inflammation [2]. The global prevalence of RA ranges from 0.25% to 1% [3], and the disease not only causes important physical disability but also severely impacts the patient’s quality of life, often resulting in bone damage, joint deformity, and potential long-term disability. Chronic medication regimens, coupled with RA’s systemic effects, pose considerable challenges in treatment and management, placing a heavy burden on both the physical and mental well-being of those affected.

### Pathogenesis

RA can affect people of all ages, although those over 40 are particularly susceptible. Despite extensive research, the causes and pathogenesis of RA remain inconclusive. Generally, genetic and environmental risk factors are regarded as the contributors to disease development [4]. Key genetic risk factors, especially shared epitopes (SEs) of specific human leukocyte antigen (HLA) class II antigens, such as the HLA-DR beta1 (HLA-DRB1) locus, are closely associated with RA development by influencing T cell responses to self-antigens [5]. Environmental factors, including exposure to dust, obesity, smoking, imbalanced gut microbiota, and periodontal disease, have also been identified as contributors to the risk of developing RA, potentially influencing both the onset and persistence of the disease [6,7].

In line with the nature of autoimmune diseases, the autoimmune response in RA often precedes clinical symptoms by years. This phase, referred to as the “pre-RA” period, can last less than a year to over a decade. During this stage, patients may show no symptoms or only subtle articular signs, yet there is often an increasing level of autoantibodies, like rheumatoid factor (RF) and anti-citrullinated protein antibodies (ACPAs) [8,9]. However, many patients test negative for conventional autoantibodies RF or ACPA [10,11]. While biomarkers such as the erythrocyte sedimentation rate (ESR) and C-reactive protein (CRP) have been incorporated into updated diagnostic criteria, these methods still have limitations [12–14]. Consequently, identifying more specific biomarkers or refining diagnostic techniques continues to remain a challenge within the realm of RA [15].

Currently, many hypotheses regarding the pathogenesis of RA are developed based on ACPA- or RF-positive cases. The discussions in this review primarily reflect the prevailing perspectives on the pathology of RA. The production of these autoantibodies gradually triggers the collapse of the individual’s self-tolerance, leading to dysfunction in a variety of immune cells and signaling networks [16]. Ultimately, this results in tissue and organ damage, particularly in those parts rich in autoantibodies, such as the joints, lungs, or blood vessels.

After an extended period of symptom-free autoimmunity, RA progresses to its subsequent stage characterized by the migration of innate and adaptive immune cells into the inflammation tissue that are driven by chemokines. This results in a gradual clinical presentation of tissue inflammation. In RA patients, T cells that are influenced by inadequate DNA repair [17] undergo aberrant differentiation, favoring the development into highly proliferative, tissue-invasive, and pro-inflammatory effector cells. These effector cells, upon invasion of the synovium, lead to severe synovial inflammation [18,19]. The synovium is a thin layer of mesenchymal tissue that runs along the joint canal under normal physiological conditions. However, in response to inflammatory stimuli, it undergoes pathological hypertrophy, marked by the uncontrolled expansion of fibroblast populations and the accumulation of more extracellular matrix (ECM) constituents. As a result, the synovial membrane becomes significantly thickened and highly vascularized. Within the subintimal layer, various immune cells, including neutrophils, macrophages, and lymphocytes, accumulate and play a critical role in driving the chronic inflammatory cascade [20].

### Clinical treatment

The clinical manifestations of RA include redness, swelling, deformity, and joint pain, especially in the synovial joints. To avoid misdiagnosis with other forms of arthritis, the clinical community relies on the classification criteria established by the American College of Rheumatology (ACR) and the European Alliance of Associations for Rheumatology (EULAR) to accurately diagnose and categorize RA [2,13]. These criteria are primarily based on the quantification and distribution of swollen joints, the serological assessment of autoantibody presence, and the temporal profile of symptomatology. In addition to clinical evaluations and serological tests, imaging methods such as magnetic resonance imaging, ultrasonography, and x-ray are employed to further detect inflammation and bone damage [21].

Early pharmacological intervention is essential for successful treatment following the onset of clinical symptoms [22]. Current treatment strategies for RA, as advised by the ACR and EULAR, address the condition from 2 angles: symptom relief by using nonsteroidal anti-inflammatory drugs (NSAIDs) and glucocorticoids (GCs), and disease modification with disease-modifying antirheumatic drugs (DMARDs) [23]. In the acute stage, NSAIDs and GCs are prescribed to alleviate pain and inflammation. However, their use is often associated with marked side effects [24,25]. DMARDs are employed to induce remission, quell autoimmune responses, and mitigate or forestall the deterioration of joint structures. The 3 main categories of DMARDs are conventional synthetic DMARDs (csDMARDs), biological DMARDs (bDMARDs), and targeted synthetic DMARDs (tsDMARDs) [23].

csDMARDs are the traditional synthetic disease-modifying drugs with unclear mechanisms. They have been widely used in clinics for years due to their efficacy and low cost. Leflunomide, hydroxychloroquine, and methotrexate (MTX) are most commonly used to treat RA. MTX often serves as the foundational or anchor drug for initial therapy owing to its relative safety and efficacy [26,27]. While MTX is regarded as the safest medication among csDMARDs, its potential side effects such as gastrointestinal toxicity, pulmonary toxicity, and hepatotoxicity are hard to overlook [28].

bDMARDs represent a newer treatment option for RA, providing targeted interventions within the immune system. These biologics work by inhibiting inflammatory pathways or suppressing the activity of immune cells, including macrophages, T cells, and B cells. However, bDMARDs are highly immunogenic, implying that the body’s immune system may recognize these drugs as foreign invaders and generate antibodies specifically targeting the medication. Additionally, bDMARDs come with certain risks, including an increased susceptibility to infections and a higher risk of tumors [29].

tsDMARDs are a class of medications developed following the advent of bDMARDs, designed to overcome some of the limitations associated with bDMARDs. These drugs typically consist of small-molecule inhibitors that target cytokine signaling pathways, with specific molecular targets such as Janus kinase (JAK) inhibitors [30,31]. The development of tsDMARDs offers new therapeutic options for RA, especially for people who are intolerant to or do not respond adequately to csDMARDs or bDMARDs. However, tsDMARDs also carry certain risks and challenges, including the potential to increase the risk of infections, cardiovascular issues, thrombosis, and the development of malignancies [32].

### Development of biomaterials in RA treatment

Although current pharmaceutical therapies have achieved success in alleviating the symptoms of RA and decelerating the disease’s progression, they often face challenges such as insufficient efficacy, poor solubility, a wide range of adverse effects, and considerable variability in individual responses. In response to clinical demands for drug delivery, biomaterials with customizable, processable, and modifiable properties have been designed for advanced drug delivery systems. These systems have shown great potential in effectively enhancing the utility of therapeutic agents, including antibodies [33], peptides [34], small molecules [35], and nucleic acids [36], offering promising solutions for RA treatment [37].

In recent years, there has been intense investigation into biomaterials for drug delivery via various methods. Different forms of biomaterials such as polymers, carbon nanotubes, hydrogels, and nanoparticles (NPs) and material compositions have been developed to solve crucial problems in drug delivery through intravenous, intraperitoneal, oral, intra-articular, and transdermal delivery. For instance, Gupta and colleagues [38] have summarized the application of nanotechnology in RA drug delivery, demonstrating how nanoemulsions, lipid NPs, liposomes, ethosomes, transferosomes, NPs, dendrimers, and polymeric micelles effectively encapsulate and deliver drugs to the site of action to exert their therapeutic effects. Peppas and colleagues [39] have made substantial contributions to hydrogel-based drug delivery systems, particularly in addressing the issues of drug specificity and retention time. They proposed that hydrogel systems can be engineered to improve the specificity and duration of drug action by tailoring their physical and chemical properties to meet specific therapeutic needs. Stem cells possess unique immunoregulatory capabilities and regenerative differentiation potential. In the treatment of RA, they can modulate the immune response, inhibit inflammation, and promote osteogenesis, offering marked application potential. Ding and colleagues [40] discussed the application of mesenchymal stem cells in RA treatment and summarized the strategies in which mesenchymal stem cells are involved. Biomaterials, as mesenchymal stem cell carriers, can enhance cell survival, improve cell retention, and regulate stem cell behaviors, greatly promoting the therapeutic efficacy of stem cells. Dhanka and colleagues [41] have discussed intra-articular drug delivery strategies using biomaterials to reduce and circumvent systemic toxicity, while Yu and colleagues [42] have reviewed the strategies for achieving precise drug delivery to targeted functional cells. The primary objectives of these biomaterials are (a) to use the material form to load and protect drugs, (b) to utilize the size and surface functionality of the materials to actively or passively deliver drugs to the required sites, and (c) to exploit the material structure for sustained release or smart responsive drug delivery. Developing biomaterial-based delivery systems to improve the effectiveness and efficiency of drugs has become a mainstream research area in RA treatment, further advancing the application of biomaterials in RA.

However, there are still notable drawbacks to using biomaterials for drug delivery. Therapeutic outcomes remain largely dependent on the efficacy of the drug itself. Even with the development of delivery systems designed for optimal drug release, the effectiveness of RA treatment is still closely tied to the potency of the pharmaceutical agents. Additionally, RA is a multifactorial and highly dynamic disease, with marked differences between patients, which cannot be adequately addressed by drugs with a single target. Furthermore, drug resistance and side effects cannot be entirely mitigated by biomaterials alone. To date, no curative treatment for RA has emerged. Therefore, new approaches and directions are urgently needed, extending beyond achieving more efficient drug delivery. The limitations of drug delivery and the complexity of the RA immune microenvironment have prompted researchers to gradually shift focus from drug delivery systems to material-based regulation, particularly the regulation of the RA immune microenvironment. Biomaterials are increasingly recognized not only as adjuncts to drug function but also as central components in RA treatment, with the potential to directly or indirectly influence the immune environment.

**Fig. 1.**
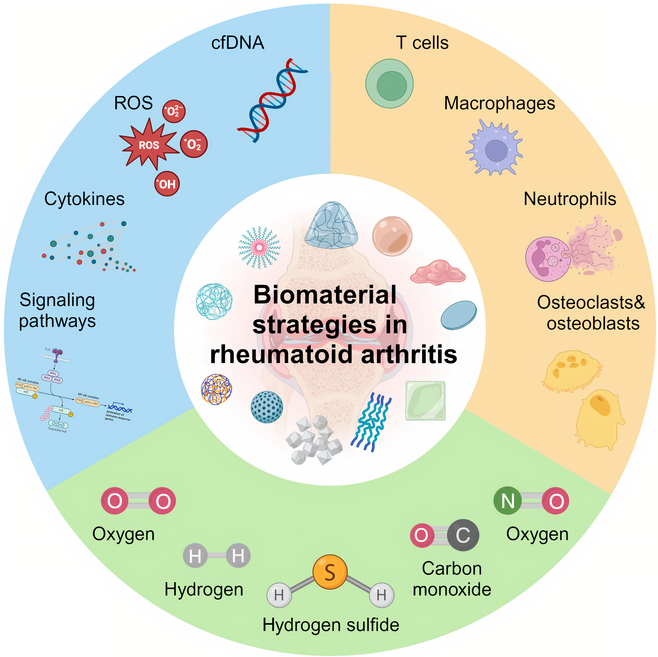
Schematic representation of biomaterials for microenvironment modulation in RA therapy.

In this review, we will initially delve into the composition of the RA immune microenvironment, focusing on the key immune cells involved in adaptive and innate immunity, as well as critical endogenous molecules, cytokines, and signaling pathways. These elements contribute substantially to the progression of inflammatory responses and the subsequent joint erosion. Subsequently, we will discuss the design of novel biomaterials for treating RA, specially targeting these immune microenvironments from 3 perspectives: cell modulation, biofactors and signaling pathway adjustment, and gas regulation (Fig. 1). For each aspect, a detailed analysis of the design principles, effects, and potential applications of these materials will be provided. A summary of the biomaterials and their targeted immune microenvironments is presented in Table 1. Through these discussions, we aim to offer new perspectives on RA treatment and guide future research and application of biomaterials.

**Table 1.** Novel biomaterial strategies for treating RA by regulating the immune microenvironment

Biomaterial category	Constituents	Design	Aim	Ref.
Nanoparticle	CeO_2_–Pt nanozyme and periodic mesoporous organosilica (PMO), indocyanine green (ICG)	A Janus nanoparticle, resembling a peanut, features CeO_2_–Pt nanozymes on one hemisphere and PMO on the other side, incorporating NIR dye ICG within its structure	ROS scavenging	[219]
Hydrogel	Tannic acid (TA), polymeric phenylboronic acid (pPBA)	The pPBA-TA nanogel was synthesized via the bonding of PBA units with TA’s catechol groups in a self-assembly process.	ROS scavenging	[281]
Hydrogel	Triptolide (TPL), PBA-modified sodium alginate (PBA-SA), inorganic composite nanoparticles	A dual dynamically cross-linked sodium alginate hydrogel	ROS scavenging	[218]
Nanoparticle	Poly-l-lysine, polyethylene glycol (PEG)-modified polypeptide with guanidine side chains	Charge-reversal polyion complex vesicles formed by combining a cationic PEGylated polypeptide featuring guanidine moieties with an anionic poly-l-lysine derivative modified by cis-aconitic anhydride	cfDNA scavenging	[282]
Nanoparticle	Oligolysine, PEG, exosomes	Exosomes with oligolysine and MMP-cleavable PEG on the membrane	cfDNA scavenging	[283]
Nanoparticle	Polycaprolactone (PCL), poly(glycidyl methacrylate) (PGMA)	A series of di-block copolymers PCL-b-PGMA with hydroxyl groups and different types of amino groups	cfDNA scavenging	[220, 284,285]
Nanoparticle	Polyarginine, thioketal, PEG	Polyarginine nanoparticles with PEG shells formed by self-assembly of amphiphilic triblock polymer mPEG-TK-PArg-b-PAsp(DIP&BZ)	cfDNA scavenging	[223]
Nanoparticle	PCL, polyamidoamine (PAMAM)	PCL serves as the degradable core, with various PAMAM dendron generations grafted as side chains.	cfDNA scavenging	[286]
Hydrogel	Deoxyribonuclease I (DNase I), third-generation polylysine dendrimer (G3K)	Cationic nanogels were synthesized through π-π stacking with 2,2′-bipyridine-4-carboxylic acid (BPY)-modified 3rd-generation lysine dendrimers, and DNase I was attached to 3 sites on the G3K10 cfDNA scavenger.	cfDNA scavenging	[287]
Nanoparticle	Neutrophil membrane, PLGA	The neutrophil membrane was coated onto PLGA polymeric cores.	Neutralize proinflammatory cytokines	[228]
Nanoparticle	Phenylalanine-grafted chitosan, cucurbit[8]uril, macrophages	Intracellularly gelated macrophages with well-preserved cell membrane structures	Neutralize proinflammatory cytokines	[229]
DNA origami	7249-Nucleotide circular single-stranded scaffold DNA (M13), CD95 ligand	The DNA origami was crafted to arrange CD95L in a hexagonal, 2D formation, with roughly 10-nm gaps between molecules, complementing the layout of membrane-bound CD95 receptor clusters.	Activation of CD95 death-inducing signaling	[231]
Nanomotor	Mg, hyaluronic acid (HA), poly(lactide-co-glycolic acid) (PLGA)	Mg microparticles coated with HA and PLGA	H_2_ production	[261]
Hydrogel	N, N-(2-Amino-1,4-phenylene) dipentyn-4-amide (DA-NOCCL), azide-functionalized HA, azide-functionalized PEG–PLA block copolymer	The PEG-PLA copolymer self-assembled and crosslinked with DA-NOCCL and HA-N_3_ to create a hydrogel.	NO scavenging	[236,288]
Hydrogel	Thiol-modified HA, CTA, NAPA	A hydrogel was formed by merging thiol-modified HA with a dual-gas crosslinker through a metal-free click reaction.	NO scavenging; H_2_S release;	[289]
Hydrogel	Mesoporous manganese cobalt oxide nanozyme, amino group (-NH_2_)-rich ε-polylysine, hydrazide group (-CONH-NH_2_) and aldehyde group (-CHO)-functionalized HA	A hydrogel inspired by biological metabolism, crafted from a dynamically cross-linked natural polymer and infused with MOF-derived nanozymes	O_2_ production; ROS scavenging	[257]
Nanoparticle	Ferrihydrite (Fh), polyvinylpyrrolidone (PVP)	Ultrasmall Fh nanoparticles were synthesized with high dispersion and coated with PVP.	O_2_ production; ROS scavenging	[258]
Molecular probe	Donor-acceptor type fluorophore containing bipyridine, carbonyl manganese	Synthesis of a novel molecular probe comprising a long-wavelength-emitting aggregation-induced emission core and a ROS-responsive manganese carbonyls cage group	ROS-triggered release of CO gas	[264]
DNA origami	M13, folic acid	A folic acid-modified triangular DNA origami nanostructure (FA-tDONs)	NO scavenging; ROS scavenging; macrophage polarization regulation	[237]
Nanomicelles	PEG, N-(2-aminophenyl) acrylamide hydrochloride (NAPA), trithiocarbonate chain transfer agent (CTA)	The block copolymer featured a cysteine-activated H_2_S donor (CTA) and NO-responsive NAPA in its core.	NO scavenging;H_2_S release; ROS scavenging; macrophage polarization regulation; impress inflammation by NF-κB signaling pathway	[251]
Nanoparticles	Au, Ag, ZnS, HA	Au@Ag colloidal particles serve as the core, doped with ZnS as a heterogeneous shell and subsequently encapsulated by HA.	H_2_ production; promote apoptosis of fibroblast-like synoviocytes (FLSs)	[262]
Hydrogel	Calcium peroxide (CaO_2_), hematoporphyrinmonomethyl ether (HMME), PLGA-PEG-PLGA	O_2_/Ca^2+^-supporting phototherapy microspheres were made and added into the aqueous PLGA-PEG-PLGA to form a hydrogel	O_2_ production; impress FLSs proliferation	[290]
Hydrogel	Phenylboronic acid grafted polylysine (PP), oxidized dextran (OD), selenium nanoparticles (SeNPs)	A hydrogel with 3 networks based on hydrogen bonds, phenylboronate ester, and Schiff base	ROS scavenging; impress inflammation by PI3K/AKT/NF-κB and MAPK pathways	[230]
Hydrogel	Sulfated HA (sHA)	sHA gel was prepared.	ROS scavenging; macrophage polarization regulation	[217]
Nanoparticle	PEGylated Ag NPs; folic acid (FA)	PEGylated Ag NPs decorated with FA	ROS scavenging; macrophage polarization regulation	[266]
Nanosheet	Fe/BiOCl, cetyltrimethylammonium bromide (CTAB)	An iron-doped piezoelectric nanosheet Fe/BiOCl with a positive charge on the surface provided by CTAB	ROS scavenging; macrophage polarization regulation	[271]
Nanosheet	Mn^III^ meso-tetrakis (4-carboxyphenyl) porphyrin, Zn^2+^	2D metal-organic framework (MOF) synthesized by benzoyloxys of MnTCPP and Zn^2+^	ROS scavenging; regulating macrophages polarization	[291]
Hydrogel	Adamantane hyperbranched polylysine, cyclodextrin-modified HA	The hydrogel was formulated with β-cyclodextrin-modified hyperbranched polylysine (HBPL-CD) and adamantane-modified hyaluronic acid (HA-Ad).	cfDNA scavenging; impress abnormal neutrophil	[277]
Hydrogel	black phosphorus nanosheets (BPNs), platelet-rich plasma (PRP), chitosan	Combined BPNs into the platelet-rich plasma-chitosan thermo-responsive hydrogel	Remove hyperplastic synovial cells and promote osteogenesis	[292]
Nanoparticle	Mesenchymal stem cell nanovesicles (MSCNVs), CeO_2_	Functionalized maleimide Ce NPs were attached using the thiol-maleimide process onto the surface of thiol-functionalized MSCNVs to form a hybrid nanosystem.	ROS scavenging; macrophage polarization regulation	[280]
Nanocatalytic material	CeO_2_, MgAl-layer double hydroxide (LDH)	CeO_2_ nanoparticles load on MgAl-LDH through electrostatic adsorption	ROS scavenging; dephosphorylation of NF-κB; macrophage polarization regulation; inhibit the formation of osteoclasts	[293]
Nanoparticle	Lentinan (LNT); Se	LNT-Se nanoparticles synthesized by mixing Na_2_SeO_3_ solution with LNT solution	ROS scavenging; regulate macrophage polarization; reduce osteoclastogenesis	[273]
Nanoparticle	Gold (Au), FA, IL-4, methylprednisolone (MP)	The Au nanocages were decorated with PEGylated FA and recombinant IL-4, and MP was encapsulated within their nanopores, resulting in IL-4@AuNCs.	Regulate macrophages polarization	[269]
Nanoparticle	BaTiO_3_ (BTO)	BTO nanoparticles, synthesized via the solvothermal method	Repair of macrophage barrier	[294]
Hydrogel	Tyramine, alginate	Hydrogel is formed by conjugating tyramine with alginate and then treated with oxygen plasma.	Construct a cartilage cell-enhancing microenvironment; regulate macrophage polarization	[295]
Nanoparticle	Zinc–curcumin complex, Cu-engineered mesoporous silica nanoparticle	Nanoparticles with a Zn-Cur core formed by the chelate interaction between curcumin and Zn^2+^ and a hybrid framework formed with copper silicate as a shell	Synergistically promote bio-mineralization based on bone marrow stromal cells	[274]

## Immune Microenvironment in RA

RA is characterized by a profoundly altered immune microenvironment, where multiple immune components are dysregulated, contributing to the chronic inflammation and tissue destruction seen in the disease. This dysregulation is not limited to any single aspect of the immune response, but rather involves complex interactions between adaptive immunity, innate immunity, DAMPs, and aberrant signaling pathways (Table 2).

**Table 2.** Key components of the RA immune microenvironment

Category	Key components	Main function/role in RA
Adaptive immune cells	CD4^+^ T cells (T_H_1, T_H_2, T_H_17, T_reg_, T_PH_, etc.)	Assist other immune cells and drive the production of autoantibodies; undergo dysregulated differentiation and secrete a variety of pro-inflammatory cytokines
	CD8^+^ T cells	Induce nearby cells to produce inflammatory cytokines and exert pro-inflammatory effects; promote the formation of germinal centers
	B cells	Produce autoantibodies; present antigens; secreting harmful cytokines
	Dendritic cells (cDCs, pDCs, moDCs)	Promote T and B cell activation; produce IFNs that enhance the production of autoantibodies; increase the production of pro-inflammatory cytokines and induce the expansion of T_H_17 cells
Innate immune cells	Neutrophils	Massively infiltrate into the site of inflammation; enhance migratory capacity; increase survival; heighten pro-inflammatory capabilities; promote the release of NETs to produce antoantibodies
	Macrophages (M1, M2)	M2/M1 imbalance; produce matrix-degrading enzymes and attract and hyperactivate pro-inflammatory T cells; macrophage barrier breakdown
Other cells	Fibroblast-like synoviocytes	Transform them into aggressive RA-FLSs; create a hypoxic environment and increase the demand for metabolites; facilitate the migration of immune cells into the synovial tissue and exacerbate hyperplasia; produce inflammatory chemokines and cytokines
	Osteoclasts	Secrete protons and proteases to demineralize cartilage and subchondral bone; expose the marrow space for synovial tissue
DAMPs	cfDNA	Triggers severe inflammation in tissue and blood
	ROS	Lead to lipid peroxidation, protein oxidation, DNA damage; ferroptosis; promote pro-inflammatory factors; activate other immune cells; enhance the formation and differentiation of osteoclasts
	NLRP3 inflammasomes	High expression of NLRP3 inflammasome in rheumatoid synovium; promote inflammation and T_H_17 cell differentiation
Inflammatory cytokines	TNF	Activates endothelial cells, synovial cells, and other immune cells; promotes antibody production; facilitates cartilage degradation and bone resorption
	IL-6	Promotes the migration of neutrophils and the infiltration of monocytes; enhances the differentiation of T_H_17 and T_PH_ cells; influences osteoclastogenesis and bone metabolism; causes intra-articular angiogenesis and joint swelling;
	IL-17A	Drives pro-inflammatory actions, angiogenesis, and osteoclastogenesis;
	IL-1β	Activates macrophages; leads to increased inflammation; induces synovial proliferation; results in bone resorption and cartilage damage
Signaling pathways	The NF-κB signaling pathway	Triggers the generation of various pro-inflammatory cytokines; activates immune cell;
	The MAPK signaling pathway	Regulates the production of pro-inflammatory cytokines; contributes to the degradation of the extracellular matrix
	The JAK/STAT signaling pathway	Expression of the JAK/STAT pathway receptors is widespread across various tissues and cells; essential for a variety of physiological mechanisms

### Adaptive immune dysregulation

Adaptive immunity [43–46], also known as acquired or specific immunity, constitutes a pivotal component of the human immune system. It is distinguished by its capacity to provide a focused immune response against specific pathogens or foreign stimuli. Upon antigen recognition, the adaptive immune system generates effector and memory cells capable of eliminating pathogens through antibody production or cytotoxic mechanisms [47].

The adaptive immune response is mediated by a diverse repertoire of lymphocytes, including both B and T cell populations. Upon encountering their specific antigens, B cells differentiate into plasma cells and secrete antibodies that bind to the antigens. These antibodies serve multiple functions: They can neutralize pathogens directly, coat them to facilitate phagocytosis (a process known as opsonization), or trigger the activation of the complement system. Together, these mechanisms contribute to the elimination of foreign substances from the body. On the other hand, T cells orchestrate the adaptive immune response through various subsets. Helper T cells such as CD4^+^ T cells assist other immune cells and provide vital signals for B cell differentiation. Cytotoxic T cells (CD8^+^ T cells) specifically target and lyse cells harboring intracellular pathogens or those expressing abnormal proteins [48].

In RA, the overactivation of immune cells, particularly T cells, caused by genetic and environmental factors, along with the collapse of the immune tolerance system, signifies the dysregulation of adaptive immunity. This dysregulation results in the production of autoantibodies, the formation of immune complexes, and the activation of inflammatory immune cells, all of which contribute to chronic inflammation and tissue damage [19,49,50]. In this section, we will briefly introduce the changes in the major immune cells that contribute to adaptive immunological dysregulation in RA and the resulting impacts.

#### T cells

T cells constitute a vital role in adaptive immunity and are indispensable in the pathogenesis of RA. Although the precise pathways through which T cells trigger RA remain incompletely understood, both clinical and experimental data underscore their critical involvement in disease progression [23,51]. For instance, inhibiting T cell activation has proven effective in clinical trials, and a significant infiltration of CD4^+^ T cells has been detected in the synovial membrane [51,52]. Studies have confirmed that individuals possessing specific HLA-DR alleles are more prone to generate autoantibodies than the general population. This increased propensity is attributed to the abnormal T cell responses linked to these alleles, which may result in the breakdown of immune tolerance and the initiation of autoimmune reactions [53]. Therefore, these aberrant T cells not only lurk within the patient’s body, participating in the “pre-RA” phase for several years and exacerbating the patient’s autoimmune environment, but also, especially CD4^+^ T cells [52,54], are directly involved in driving the inflammation.

The differentiation of CD4^+^ T cells is influenced by antigens, co-stimulatory signals, and inflammatory cytokines, all of which are prevalent in RA-affected joints. As a result, CD4^+^ T cells in joints are particularly susceptible to external influences. In RA synovium, CD4^+^ T cell populations are primarily responsible for 2 key functions. First, they drive the production of autoantibodies, exacerbating the progression of RA [55]. Second, they undergo dysregulated differentiation and secrete a variety of pro-inflammatory cytokines [19,56,57]. This abnormal differentiation is fundamentally associated with impairments in the DNA repair system and the reprogramming of cellular energy metabolism [19,58], leading CD4^+^ T cells to transform into highly proliferative, tissue-invasive, and proinflammatory effector cells like T helper 1 (T_H_1), T_H_17, and peripheral helper T (T_PH_) cells, instead of differentiating into relatively quiescent memory T cells [54]. Simultaneously, the inflammatory environment inhibits differentiation into anti-inflammatory T regulatory (T_reg_) cells, resulting in an imbalance between pro-inflammatory and immunosuppressive T cell populations [59]. In addition, even the T_reg_ population present in inflamed joints faces challenges in maintaining a stable phenotype. They can differentiate into T_H_1-like, T_H_17-like, and osteoclastogenic-like T_reg_ cells, leading to impaired immune tolerance functionality [60–62]. The dysregulated balance, particularly between pro-inflammatory and immunosuppressive cells (like T_H_17 and T_reg_ [63]), promotes the further secretion of pro-inflammatory cytokines, including tumor necrosis factor (TNF), interferon-γ (IFN-γ), and interleukin-17 (IL-17) [64]. The sustained release of these cytokines perpetuates chronic inflammation, intensifying the degree of the inflammatory response in RA and driving the ongoing progression of joint inflammation and tissue destruction.

Although CD4^+^ T cells comprise the majority of synovial T cells, CD8^+^ T cells are also functionally marked. For example, specific subsets of CD8^+^ T cells have been identified as potential predictors of autoimmune arthritis severity [19,65]. However, their precise contribution to the pathology is still not entirely clear. Emerging evidence suggests that CD8^+^ T cells are involved in RA progression. Studies on human samples indicate that these cells are crucial for the formation of germinal centers, which are present in about half of RA patients, potentially contributing to both disease initiation and persistence [66]. Compared to healthy individuals, CD8^+^ T cells from RA patients more frequently express granzyme K (GzmK), with GzmK-positive CD8^+^ T cells accounting for 75% of the total CD8^+^ T cells [67]. These GzmK CD8^+^ T cells may induce nearby cells to produce inflammatory cytokines and exert pro-inflammatory effects, potentially through the activation of protease-activated receptors, such as protease-activated receptor-1 (PAR-1) or PAR-2 [68].

#### B cells

Like T cells, B cells are integral to the adaptive immune response and contribute to the development of RA [69]. While research into the exact mechanisms by which B cells contribute to RA is ongoing, the presence of classic RA markers—autoantibodies such as ACPA and RF—derived from autoreactive B cells, underscores their importance in the disease [70]. Further evidence supporting the functional significance of B cells comes from studies involving B cell depletion therapies [71–73]. For example, RA patients treated with rituximab (antibody targeting CD20, 95% expressed in human B cells) demonstrated clinical improvements, including a reduction in synovial B cells, plasma cells, and immunoglobulin G (IgG) levels, indicating that decreasing B cell counts might help suppress the disease [74]. Several B cell depletion therapies have received approval from the U.S. Food and Drug Administration (FDA) for RA treatment.

In light of long-term research findings [73,75,76], it is now understood that B cells contribute to RA in 3 primary ways: producing autoantibodies, presenting antigens, and secreting cytokines. These functions not only facilitate the progression from the “pre-RA” phase to acute synovitis but also promote inflammation within the synovium. While the focus remains on autoreactive B cells producing autoantibodies under T cell stimulation in humoral immunity, growing attention is being paid to the interaction between B cells and T cells. For instance, it has been discovered that B cells can act as antigen-presenting cells (APCs), presenting antigens to T cells and promoting T cell activation [77,78]. Besides, B cells are capable of secreting various cytokines, such as IL-6, TNF-α, IL-1β, and receptor activators of nuclear factor κB (NF-κB) ligand (RANKL), which contribute to inflammation and bone destruction [79]. In vitro experiments have demonstrated that RANKL secreted by B cells can promote the differentiation of monocytes into osteoclasts [75]. Moreover, analysis of synovial tissue has revealed various activated B cell subsets, indicating that B cells within the synovium might promote the formation of tertiary lymphoid tissues. These structures support the local generation of antibody-secreting B cells and may also play an antibody-independent role by producing proinflammatory and osteoclastogenic cytokines [67,80].

#### Dendritic cells

As their name suggests, dendritic cells (DCs) are equipped with dendritic protrusions resembling branches, which facilitate their role as APCs by enabling effective antigen capture, processing, and presentation. DCs are traditionally categorized within the realm of the innate immune system. However, they are strategically positioned at the interface of these 2 arms of the immune system, capable of integrating signals from the innate immune system and initiating specific adaptive immune responses. In RA, immature DCs in peripheral tissues differentiate in response to excessive pro-inflammatory cytokines, autoantibody-containing immune complexes, and endogenous cytokines or damage-associated molecular pattern molecules (DAMPs) [55,81]. These differentiated DCs then migrate to the lymph nodes via the lymphatic system and process antigens into major histocompatibility complex (MHC) molecules [82]. This antigen presentation activates naïve T cells, promoting their differentiation into various T cell subsets [83]. RA pathogenesis involves 2 major DC subsets: conventional DCs (cDCs including cDC1 and cDC2) and plasmacytoid DCs (pDCs), both of which are found in synovial joints with high concentrations [84,85]. cDC1 and cDC2 specialize in presenting antigens via MHC-I and MHC-II to T helper cells, thereby promoting T and B cell activation. At the same time, pDCs typically accumulate in the peripheral organs, where they produce IFNs that enhance the production of autoantibodies [86]. However, some studies suggest that pDCs may also play a protective role in RA development, as depletion of pDCs has been shown to exacerbate arthritis [87]. Therefore, further research is required to explore the precise processes and roles of pDCs in RA.

Synovial membranes in RA are home to cDCs and pDCs, as well as a substantial number of monocyte-derived DCs (moDCs). These moDCs have been shown to contribute to the pathogenesis and progression of RA by increasing the production of pro-inflammatory cytokines and inducing the expansion of T_H_17 cells. Thus, the presence of cDCs and moDCs contributes to the inflammatory process in multiple ways: by presenting antigens to activate T and B cells, secreting inflammatory factors that intensify local inflammation, and releasing chemokines that draw more immune cells, such as monocytes, neutrophils, and macrophages, to the site of inflammation [88–90].

### Innate immune overactivity

The pathological characteristics of RA are primarily centered around systemic joint inflammation, which manifests as joint swelling and pain. This inflammation is propelled by intricate interactions within the innate immune system, encompassing immune cells like monocytes, DCs, macrophages, and neutrophils, as well as synovial resident cells such as fibroblast-like synoviocytes (FLSs) and osteoclasts (OCs) [91]. These innate immune cells constitute the body’s initial defense, serving as phagocytes, antigen-presenting entities, and cytokine generators, playing a crucial role in both the onset and continuation of the disease. In particular, the activation of innate immune cells in the synovium triggers a cascade of events, including the recruitment and activation of FLSs and OCs, which work in concert with immune cells to drive the pathogenesis of RA. While the initial breakdown of self-tolerance may stem from erroneous antigen recognition by APCs, the clinical features observed in the later stages of RA are mainly caused by the combined actions of these innate immune cells and the synovial cells they interact with, including FLSs and OCs [92]. Here, we will provide a more detailed explanation of how these key cells influence RA pathology, emphasizing their contributions to the disease’s progression.

#### Neutrophils

Neutrophils constitute the majority of white blood cells in circulation and serve as the first line of defense against inflammatory or infectious challenges. As effector cells of the innate immune system, neutrophils exhibit a swift response to the incursion of bacteria, fungi, and other pathogens in infected tissues [93]. These cells possess robust phagocytic capabilities and can secrete high levels of cytotoxic products. They recognize pathogens through pattern recognition receptors (PRRs), engulf them, and eliminate them [94]. Additionally, neutrophils are equipped to release a plethora of cytotoxic agents such as reactive oxygen species (ROS), myeloperoxidase (MPO), numerous hydrolytic enzymes, and antimicrobial peptides, thereby reinforcing the body’s antimicrobial arsenal [95].

Under normal homeostatic conditions, neutrophils have a short lifespan, undergoing rapid apoptosis after eliminating pathogens and being phagocytosed by macrophages that are used to “clean up” the battlefield [96,97]. These macrophages release a range of anti-inflammatory signals that prevent further infiltration of neutrophils, thereby suppressing the inflammatory response. However, in RA, neutrophils massively infiltrate into the sites of inflammation [98,99]. Influenced by the inflammatory environment and immune complexes, these neutrophils exhibit functional abnormalities, including enhanced migratory capacity, increased survival, and heightened pro-inflammatory capabilities [100–102]. These dysregulated neutrophils play a crucial role in both the initiation and perpetuation of RA. Specifically, activated neutrophils have an increased tendency to spontaneously form neutrophil extracellular traps (NETs) [103]. NETs are extracellular fibrous networks composed of DNA, MPO, neutrophil elastase (NE), matrix metalloproteinases (MMPs), histones, and granule proteins, which are designed to capture and eliminate invading pathogens and are subsequently degraded and cleared [104]. However, in the pathological state of RA, NET degradation is inhibited, leading to persistent NET formation by activated neutrophils in an imbalanced inflammatory environment [105]. Furthermore, the formation of NETs is closely associated with the pro-inflammatory milieu in RA [106]. In vitro experiments have indicated that neutrophils exposed to the serum of RA patients, particularly those who are ACPA- or RF-positive, along with pro-inflammatory cytokines like IL-17A and TNF-α, are readily induced to form NETs. Moreover, compared to neutrophils from healthy individuals, those from RA patients exhibit a greater propensity to form NETs [107]. NETs contain a plethora of cytotoxic components released by neutrophils, including ROS, pro-inflammatory factors, immune-stimulating proteins, and autoantigens, such as citrullinated proteins, that perpetuate autoimmune reactions. These toxic substances can not only impact local immune cells and synovial cells, leading to exacerbated inflammation, pannus formation, and joint damage, but the proteins they secrete, such as citrullinated proteins and carbamylated proteins, also serve as part of autoantigens, further intensifying the progression of RA [103,108–110]. Concurrently, antibodies against these antigens such as ACPAs stimulate the release of peptidylarginine deiminase (PAD) from neutrophils, facilitating the modification of arginine proteins into citrullinated proteins by neutrophils, thus creating a vicious cycle of autoantibody production [111]. As a result, the abnormal accumulation and functional alterations of neutrophils in RA greatly contribute to the persistence of inflammatory responses and the progression of the disease.

#### Macrophages

The RA pathogenesis involves the activation of innate immune cells, with macrophages being pivotal. They initiate injury and mitigate it by fostering inflammation and later resolving it, aiming to reestablish tissue immune equilibrium [112,113].

A dynamic interplay exists within joints between self-renewing tissue-resident macrophages (STMs) and circulating monocytes that infiltrate into the joint in response to chemokines and inflammatory factors, differentiating into macrophages upon arrival [114,115]. These macrophages are traditionally classified into 2 distinct groups based on their functions and metabolic pathways: “M1-like” macrophages, which are pro-inflammatory, and “M2-like” macrophages, which are associated with tissue repair and anti-inflammatory effects [116]. The expression of inducible nitric oxide synthase (iNOS) is a defining characteristic of classically activated macrophages, referred to as M1 macrophages [117]. They highly express MHC II, CD80, CD86, CD38, and Toll-like receptor 4 (TLR4) and secrete pro-inflammatory cytokines such as IL-1β, IL-6, and TNF-α, as well as chemokines such as C-C motif chemokine ligand 7 (CCL7) [118]. M1 macrophages contribute to cartilage damage by producing matrix-degrading enzymes and attracting and hyperactivating pro-inflammatory T cells. In contrast, M2 macrophages express arginase, which metabolizes l-arginine to urea and l-ornithine, promoting tissue healing. They produce anti-inflammatory cytokines such as transforming growth factor-β (TGF-β) and IL-10, clear debris, and promote tissue repair while activating regulatory T cells to further support anti-inflammatory processes [117–122]. The balance between M1 and M2 macrophages is marked in RA pathology, with M1 macrophages often predominating over M2. Notably, the phenotypes of STMs in RA patients are diverse, with some exhibiting a mixed phenotype that deviates from the strict M1 or M2 profiles. This heterogeneity underscores the importance of advancing our understanding of macrophage phenotypes in RA research, as it has profound implications for disease management and treatment strategies [123,124].

In RA, the chronicity of tissue inflammation and repair is governed by intricate interactions between resident and infiltrating macrophages in the affected tissues. STMs, which originate from embryonic precursors, form an immune-protective lining barrier in healthy synovium to restrict inflammatory reactions [125–128]. However, during RA, the distortion of this protective barrier and its breakdown allow tissue-infiltrating macrophages to become predominant. These infiltrating macrophages are marked contributors to the pathology of RA by producing pro-inflammatory cytokines such as TNF and IL-6, chemokines such as C-C motif chemokine ligand 2 (CCL2), and small-molecule mediators of inflammation such as ROS [125,129,130].

#### Fibroblast-like synoviocytes

The healthy synovial membrane consists of STMs and FLSs, a type of mesenchymal cells, which form a lining up to 4 cell layers thick through cell–cell contacts [129,131]. FLSs are fundamental to maintaining joint homeostasis by supplying the joint cavity and contiguous cartilage with nutrients and lubricating molecules, including hyaluronic acid and lubricin [132]. They maintain the balance of ECM deposition, assembly, destruction, and removal, primarily by producing matrix-degrading enzymes such as cathepsins and MMPs [131].

In the RA joint, FLSs undergo stable activation induced by cytokines released from T cells and macrophages, resulting in long-term phenotypic alteration that transforms them into aggressive RA-FLSs. These changes include enhanced attachment to the ECM, increased invasiveness, and resistance to apoptosis mediated by cell surface receptors. Elevated expression of adhesion molecules, such as cadherins and integrins, promotes the invasive behavior of RA-FLSs and their binding to cartilage [131,133,134]. The synovium can become dramatically hyperplastic, with the number of cell layers in the intimal layer increasing up to 20-fold [131,134]. Synovial hyperplasia and immune cell infiltration create a hypoxic environment and increase the demand for metabolites, triggering angiogenesis. This process further facilitates the migration of immune cells into the synovial tissue and exacerbates hyperplasia [135]. The pannus, which is the leading edge of the expanding synovial tissue at the cartilage–bone interface and is primarily composed of macrophages, osteoclasts, and FLSs, envelops the cartilage and erodes into the bone, leading to the formation of empty lacunae and depriving cartilage of its ability to replenish the matrix [132]. Beyond direct tissue damage, RA-FLSs engage in inflammatory signaling by producing essential chemokines and cytokines, including TNF and IL-7, fostering the migration, activation, and survival of T and B cells, aggravating oxidative stress through excess ROS production, and expressing RANKL, a key cytokine mediator of osteoclast differentiation and bone resorption [136–138].

#### Osteoclasts

In RA, the activation of fibroblasts and macrophages in synovial tissue is crucial for bone erosion. Activated fibroblasts secrete RANKL and macrophage colony-stimulating factor 1 (M-CSF, also known as CSF-1), which induce OC differentiation [139,140]. OCs, the bone-resident macrophages, possess a distinct ability to destroy bone, playing important roles in both normal bone remodeling and pathological bone erosion in RA [140–142].

Mature OCs secrete protons and proteases to demineralize cartilage and subchondral bone, degrading the bone matrix [137,143,144]. The external attack by osteoclasts could potentially penetrate the layer and expose the marrow space to synovial tissue in RA. The inflamed synovial tissue can breach the cortical bone barrier, leading to direct exposure of the underlying bone marrow to inflammatory infiltrates. This, in turn, results in the replacement of fat-rich bone marrow with a B cell-enriched mononuclear cell aggregate [145]. Since there are minimal signs of repair in eroded periarticular bone, it is important to promptly regulate the inflammation to prevent further damage or its progression.

### DAMP dysregulation

DAMPs are small molecules released by stimulated or damaged cells and tissues. They can be categorized into ECM DAMPs, intracellular protein DAMPs, metabolic DAMPs, and nucleic acids DAMPs [146]. PRRs recognize these molecular patterns due to their unique locations or biochemical properties, which trigger further inflammation and immune responses [147]. In the context of RA, DAMPs become dysregulated due to oxidative stress and the destruction of cells and tissues in the joints, which are intricately linked to the pathophysiology of the disease.

#### Circulating-free DNA

Under normal conditions, DNA is located intracellularly. However, various cells such as neutrophils, macrophages, lymphocytes, monocytes, and FLSs undergo apoptosis, necrosis, pyroptosis, ferroptosis, or NETosis because of the abnormal physiological environment in RA patients, causing the release of DNA into the bloodstream to form circulating-free DNA (cfDNA). cfDNA is mainly composed of double-stranded nuclear DNA (nDNA) and mitochondrial DNA (mtDNA) [148]. The release of cfDNA in RA is also associated with the proliferation of rheumatoid synovial cells, which can spontaneously release cf-nDNA into the circulation [149]. The levels of cfDNA in synovial fluid and serum of RA patients are higher than in healthy individuals [150,151].

The cfDNA in synovial fluid from patients with RA is enriched with hypomethylated cytosine–phosphate–guanine (CpG)-motif-containing sequences, which trigger severe inflammation both in vivo and in vitro [152]. The inflammation is induced via TLR9, a receptor for DNA sequences containing unmethylated CpG motifs. TLR9 can be activated by cfDNA, which further activates NF-κB and interferon-regulatory factor-7 (IRF-7) through the myeloid differentiation primary response gene 88 (MyD88)-dependent pathway [148]. NF-κB is one of the main inflammation pathways in RA and is linked to bone erosion [153]. Moreover, cfDNA can activate human polymorphonuclear neutrophils (PMNs) and aggravate inflammation through a TLR9-dependent way [154]. The activation of TLR9 by cfDNA can be mediated by high-mobility group box 1 (HMGB1) and the receptor for advanced glycation end products (RAGE), enhancing its binding and activation efficacy [155,156].

#### Reactive oxygen species

ROS are reactive species generated by the partial reduction of oxygen. These species contain superoxide anion (O_2_·^−^), hydroxyl radical (·OH), singlet oxygen (^1^O_2_), hydrogen peroxide (H_2_O_2_), peroxy radical (ROO·), and perhydroxyl radical (HO_2_·) [157–159]. ROS are produced during the normal cellular metabolism in aerobic organisms and play roles in cell proliferation, recruitment, and apoptosis [160]. In healthy individuals, ROS production is minimal and is balanced by antioxidants in the plasma [161]. However, in RA patients, cells normally execute oxidative phosphorylation and generate byproduct ROS. Lipoproteins, lipopolysaccharides, and cytokines, including TNF-α, IL-1β, and IFN-γ, activate nicotinamide adenine dinucleotide phosphate (NADPH) oxidases. Under oxidative conditions, NADPH oxidases mediate the activation of phagocytes such as macrophages and neutrophils, leading to an increase in oxygen consumption and the production of superoxide anion (O_2_·^−^), which can then transform into other ROS forms. As a result, the total generated ROS exceed the physiological buffering capacity, leading to oxidative stress [160,162]. This oxidative stress disrupts DAMP regulation and exacerbates RA pathology.

Oxidative stress in RA patients leads to lipid peroxidation, protein oxidation, DNA damage, and the production of harmful reactive substances. Lipid peroxidation can compromise cell membranes, and oxidized polyunsaturated fatty acids can generate cytotoxic aldehydes such as malondialdehyde that further damage proteins and DNA [163]. The accumulation of lipid peroxides also triggers ferroptosis [164]. ROS-induced oxidation of low-density lipoprotein (LDL) results in the production of pro-inflammatory factors, including adhesion molecules and growth factors. Meanwhile, highly oxidized LDL can be rapidly engulfed by macrophages, leading to the formation of foam cells that may contribute to the development of atherosclerosis [165].

In addition, ROS also regulate cellular functions. As a result of elevated levels of oxidative stress, T lymphocytes in the inflamed synovium of RA patients exhibit severe hyporesponsiveness to antigen stimulation. These T cells stay in the synovium for prolonged period, which contributes to the sustained presence of chronic inflammation [166].

Excessive ROS can have a destructive effect on both bone and cartilage. ROS play a role in regulating the formation and differentiation of osteoclasts, thereby contributing to bone destruction [167]. Chondrocytes synthesize glycoproteins, collagens, proteoglycans, and hyaluronan to maintain the activity of the ECM [168]. However, hypochlorite and superoxide radicals can induce the breakdown of hyaluronic acid [169], while chondrocyte lipid peroxidation mediates the degradation of cartilage matrix proteins [170]. ROS can also cleave proteoglycans at their core region and the binding sites with hyaluronan [171].

#### NLRP3 inflammasome

The NLRP3 inflammasome, a key component of the innate immune system, consists of the receptor protein NLRP3, the adapter protein ASC, which contains a caspase recruitment domain, and the inflammatory protease caspase-1 [172]. The NLRP3 inflammasome can be activated and primed by 2 classes of stimuli: pathogen-associated molecular patterns (PAMPs) and DAMPs. For instance, the transcriptional up-regulation of the NLRP3 inflammasome can be activated by cytokines such as TNF or IL-1β. Then, various PAMPs and DAMPs activate upstream signal events, such as K^+^ efflux and mitochondrial ROS production, leading to the activation of NLRP3 inflammasome [173]. Studies have confirmed the high expression of NLRP3 inflammasome in rheumatoid synovium [174]. Overproduced NLRP3 inflammasome can aggravate the symptoms of RA patients. Activated by the NLRP3 inflammasome, caspase-1 proteolytically induces the maturation and release of pro-inflammatory cytokines IL-1β and IL-18, thus promoting inflammation [175]. Meanwhile, activated caspase-1 cleaves gasdermin D (GSDMD), resulting in pyroptosis [176]. The NLRP3 inflammasome in CD4^+^ T cells promotes inflammatory T_H_17 cell differentiation by producing IL-1β [177].

### Abnormal cytokine and signal pathway

Systemic inflammation is one of the primary features of RA, characterized by abnormal signaling and the overexpression of a variety of inflammatory cytokines. Activated adaptive immune-related cells and innate effector cells form an intertwined and complex network, particularly in the joints, collectively promoting the production of pro-inflammatory cytokines and maintaining a highly inflammatory immune microenvironment within the body [138,178]. Concurrently, these pro-inflammatory factors influence the physiological activities of cells, such as activation, differentiation, migration, and immune responses, through various signaling pathways, thereby continuously intensifying the progression of RA.

#### Inflammatory cytokines

TNF is one of the most critical cytokines involved in the pathogenesis of RA [179–181]. It is initially produced in a transmembrane protein form (memTNF), which is then cleaved by metalloproteinases such as TNF-α-converting enzyme (TACE), leading to the release of soluble TNF (sTNF) [182]. TNF exerts its diverse biological activities by activating TNF receptor 1 (TNFR1) and TNFR2. TNFR1 is widely expressed on various cells and is primarily responsible for inducing inflammation and tissue degeneration, as well as mounting an immune defense against pathogens. Consequently, when TNF is overexpressed, it can mobilize virtually all cells within the body [183].

One of the primary sources of TNF is macrophages derived from monocytes. Upon secretion, TNF can further activate endothelial cells, synovial cells, and other immune cells such as macrophages and T cells through TNFR1, prompting these cells to release more pro-inflammatory cytokines like IL-6 and IL-1β. These cytokines engage in establishing an immune microenvironment in the synovium [50,184]. TNF can regulate the physiological processes of T and B cells, promoting the development of T_H_1 and T_H_17 cells and antibody production [185]. Moreover, it induces the differentiation of monocytes into osteoclasts through RANKL, facilitating cartilage degradation and bone resorption [186].

IL-6 also plays a critical role in RA development. The levels of IL-6 in the synovial fluid and serum of RA patients are significantly elevated [187]. Targeted blockade of IL-6 can markedly alleviate RA symptoms in patients [188,189]. IL-6 is predominantly produced by macrophages in response to pathogens or DAMPs associated with inflammation, triggering acute phase and immune reactions to neutralize infectious agents and repair damaged tissues [190,191]. Various stimuli, such as TLR ligands, IL-1β, and TNF, promote IL-6 transcription. The continuous production of IL-6 in synovial fibroblasts stimulated by TNF-α is a hallmark of RA [192]. IL-6 is involved in both innate and adaptive immunity and substantially contributes to the clinical manifestations of RA [193]. In innate immunity, IL-6 is responsible for the maturation of inflammatory infiltrates, promoting the migration of neutrophils and the infiltration of monocytes [188,194]. On the adaptive side, it regulates the differentiation of T_reg_, T_H_17, and T_PH_ cells by down-regulating the expression of Foxp3 through the transcription factor signal transducer and activator of transcription 3 (STAT3), thereby inhibiting T_reg_ and promoting T_H_17 differentiation [195,196]. Furthermore, IL-6 works synergistically with TNF to influence osteoclastogenesis and bone metabolism, leading to bone destruction. It is a major participant in vascular endothelial growth factor (VEGF)-induced processes, indirectly causing intra-articular angiogenesis and joint swelling [197,198].

IL-17A is recognized as a central cytokine in the pathogenesis of RA, primarily produced by T_H_17 cells [107,199–201]. The presence of IL-17A in inflamed joints [202,203], together with compelling evidence from in vitro studies and experimental arthritis models, underscores its role in driving pro-inflammatory actions [203], angiogenesis [204], and osteoclastogenesis [205,206].

IL-1β, predominantly produced by monocytes or macrophages, is another marked factor in RA. It activates macrophages and leads to increased inflammation, induces synovial proliferation, and also activates osteoclasts, ultimately resulting in bone resorption and cartilage damage.

#### Signaling pathways

The abnormal signal transduction of a variety of biofactors is a component of the progression and development of RA, which can lead to an increase in pro-inflammatory factors and the overactivation of immunity. Key pathways involved in this process include the NF-κB signaling pathway, the mitogen-activated protein kinase (MAPK) signaling pathway, the phosphatidylinositol 3-kinase/protein kinase B (PI3K/AKT) signaling pathway, and the JAK/STAT signaling pathway [207].

The NF-κB signaling pathway, composed of NF-κB, NF-κB inhibitors (IκBs), and IκB kinases (IKKs), is involved in the activation and differentiation processes of various immune cells [208]. Once activated, NF-κB can trigger the generation of various pro-inflammatory cytokines, including TNF-α, IL-1β, and IL-6, thereby accelerating RA progression [209]. Additionally, the activation of NF-κB can be reinforced by the overexpression of pro-inflammatory cytokines, resulting in a self-perpetuating loop that exacerbates the development of RA.

The MAPK pathway encompasses a series of cascade protein kinases, including mitogen-activated protein/extracellular signal-regulated kinase (MEK) and extracellular signal-regulated kinase (ERK) [210]. Typically, the MAPK signaling cascade initiates with the activation of MAPK kinase kinases (alternatively designated as MKKKs or MAP3Ks), proceeds through the intermediate layer of MAPK kinases (also referred to as MKKs, MEKs, or MAP2Ks), and culminates with the activation of MAPKs at the downstream level. This cascade modulates the function of various transcription factors, including NF-κB, which subsequently drive the expression of MMP genes, playing a key role in governing cellular processes like proliferation, differentiation, and apoptosis [211]. In RA, it regulates the production of pro-inflammatory cytokines and is important for downstream receptor signaling cascades for TNF-α, IL-1, and IL-17. It also contributes to the degradation of the ECM mediated by MMPs, which promotes cartilage destruction [212].

The JAK/STAT pathway is one of the most important routes in cytokine signal transduction, comprising 3 main components: tyrosine kinase-associated receptors, JAKs, and STATs [213]. This pathway is essential for a variety of physiological mechanisms, such as immune modulation, cellular differentiation, and metabolism. The expression of the JAK/STAT pathway receptors is widespread across various tissues and cells. In RA, the most marked inflammatory and immune responses involve the JAK/STAT pathway. The FDA has approved tofacitinib, a JAK inhibitor, as the clinical drug for RA treatment, and other JAK pathway inhibitors are in various stages of preclinical development or clinical trials, underscoring the significance of the JAK pathway in RA [214]. The approval of tofacitinib, along with ongoing research, highlights the JAK pathway as a pivotal target in the therapeutic strategy against RA, given its involvement in signaling for numerous cytokines playing a part in the disease’s pathogenesis.

Moreover, other pro-inflammatory factors contribute to RA development. Kondo et al. [215] have compiled a comprehensive table summarizing the key cytokines involved in the pathology of RA and their pathological roles. This thorough examination emphasizes the complexity of the immune response in RA, highlighting the intricacies of cytokine interactions that drive the inflammatory processes characteristic of the condition.

## Biomaterials for Regulation of Biofactors and Signaling Pathways

DAMPs, pro-inflammatory cytokines, and signaling pathways converge to orchestrate a complex cascade of immune activation and inflammation. Although distinct in their origins and specific roles, these elements share a common thread in modulating the disease’s progression by initiating and intensifying inflammatory responses. DAMPs alert the immune system to cellular damage, while cytokines facilitate communication between immune cells, further driving inflammation. In these processes, signaling pathways serve as the conduits, transducing these biofactors to either amplify or attenuate the inflammatory cascade.

As the understanding of RA’s pathological mechanisms deepens, the treatment strategies have evolved from mere symptom relief to precise regulation of biofactors and signal regulation. Biomaterials have emerged as an important strategy in this field, offering new perspectives and tools for regulating inflammatory factors and signaling pathways. Their unique biological properties and controllable physiochemical properties allow their intervention at the molecular level. An increasing number of biomaterials are being developed to intervene in the pathological processes of RA, offering targeted and precise modulation of the disease’s molecular mechanisms.

### Biomaterials for DAMP regulation

The unique intracorporeal milieu of patients with RA orchestrates the dysregulation of DAMPs. These endogenous molecules, emanating from the RA microenvironment, subsequently activate immune responses that exacerbate the progression of the disease. Consequently, the modulation of DAMPs is of significance in the therapeutic management of RA. Despite the vast array of DAMPs and the incompletely understood biological mechanisms, there remains a relative scarcity of pharmacological treatments that specifically target DAMPs. This scarcity is largely attributed to the complexity and diversity of DAMPs, which can imply multiple molecules in a single inflammatory condition, complicating the pathophysiological mechanisms. However, biomaterials have demonstrated a natural advantage in regulating certain DAMPs, such as cfDNA and ROS. Researchers have leveraged the structural characteristics and properties of cfDNA and ROS to design, synthesize, and modify biomaterials with targeted structures and components. These biomaterials are engineered to possess the capability to clear cfDNA or ROS, offering a novel approach to the treatment of RA.

The clearance of ROS is a pivotal strategy in immunomodulation for RA treatment using biomaterials [216]. These biomaterials are specifically engineered to respond intelligently to ROS, facilitating effective drug release under inflammatory conditions. As the material responds to ROS to promote drug release, ROS levels are concurrently reduced through reactions with the ROS-responsive moieties within the materials. When the materials contain enough density of these ROS-responsive groups, they will effectively acquire ROS-scavenging capabilities. The most commonly employed method involves the use of polymers or functional substances with natural antioxidant properties, such as polydopamine, hyaluronic acid, and tea polyphenols. The structural units of these materials themselves possess inherent reducing properties, which enable them to react with ROS and thereby decrease the concentration of ROS. For example, a sulfated hyaluronic acid hydrogel effectively inhibits ROS levels and improves joint cavity swelling in collagen-induced RA in rats [217]. Similarly, incorporation of ROS-responsive chemical repeating units such as phenylboronic ester bonds into materials with relatively weak intrinsic antioxidant capacity represents another important strategy. A hydrogel crosslinked by phenylboronic acid and tea polyphenols could actively respond to and clear excess ROS in the RA microenvironment, modulating joint inflammation [218]. Furthermore, the catalytic function of nanozymes can also be harnessed. For instance, a multifunctional Janus platform using cerium–platinum nanozymes for ROS clearance achieves prominent anti-inflammatory and therapeutic effects in RA treatment [219]. Based on the in-depth development of ROS regulation, ROS modulation is now increasingly being integrated as a fundamental material functionality in the combinatorial strategy for modulating the immune microenvironment in RA. This approach is gaining traction due to its potential to deliver therapeutics and actively participate in the intricate balance of immune activation and inflammation characteristic of RA.

The clearance of cfDNA can effectively suppress systemic inflammation. Electrostatic adsorption using cationic polymers is a straightforward method to clear cfDNA. Cationic NPs formed by self-assembly of a poly(lactide-co-glycolic acid) (PLGA)-block-poly(2-(diethylamino)ethyl methacrylate) (PDMA) block copolymer show a high binding affinity for cfDNA, thereby effectively alleviating inflammation [220]. These NPs, with a diameter of approximately 40 nm, effectively suppress the abnormal activities of primary synovial fluid monocytes and FLSs that are induced by cfDNA from RA patients.

Nonetheless, due to the negative charge of cell membranes, the cationic polymeric materials can induce membrane disruption in a charge-dependent manner, adversely affecting cellular biological properties [221]. These materials also prematurely interact with negatively charged macromolecules, including mucin glycoproteins, serum proteins, and proteoglycans, as well as cells with polyanionic surface structures like red blood cells. The premature engagement renders them ineffective before they reach their intended target. To address this challenge, the materials can be engineered to respond to the specific microenvironment at the target site, including pH, redox, and enzymatic activity. This can be achieved by shedding their positively shielding, high-volume side chains [such as polyethylene glycol (PEG)] or anionic components at the target location, thereby regaining their positive charge [222]. For example, PEG-TK-NP_Arg_ NPs have been innovatively developed by utilizing a ROS-responsive thioketal (TK) segment to link arginine groups with a positively shielding PEG shell [223]. In the ROS-rich environment of RA joints, the TK segment cleaves, consuming ROS and exposing polyarginine. The guanidinium groups of polyarginine form Gua/PO_4_^2-^ salt bridges with cfDNA, leading to tight binding and creating PEG-TK-NP_Arg_-cfDNA complexes where the spacing between cfDNA molecules is significantly smaller than the cavity size of TLR9. The poor spatial compatibility between NP_Arg_-cfDNA and TLR9 prevents TLR9 activation in contrast to LL37-ctDNA complexes (LL37 is an endogenous peptide, which easily self-assembles with nucleic acids). This approach offers a novel strategy for cfDNA clearance in treating RA inflammation. After cationic polymers competitively extract cfDNA from immune complexes, they also compress the distance between cfDNA molecules, inhibiting their spatial matching and activation with TLR9.

It is challenging to develop drugs that directly target DAMPs, although reducing DAMPs has long been an innovative concept in RA treatment. The advent of biomaterials has transformed this concept from a theoretical idea into a practical reality. These biomaterials typically use biocompatible molecules and are engineered into nanoscale forms that facilitate systemic distribution. They can both encapsulate drugs to create synergistic effects that improve the RA immune microenvironment (e.g., ROS-scavenging) and actively enable targeted therapy through their intrinsic properties and structure (e.g., cfDNA-clearing). This approach reduces reliance on traditional drugs, minimizes their toxic side effects, and allows for more precise targeting of therapeutic goals. It advances our understanding of DAMPs as therapeutic targets in RA and promotes the further application of biomaterials in medical and healthcare fields.

Nonetheless, it is inevitable that, despite the experimental evidence demonstrating the safety of these materials through cell and animal studies, the specific mechanisms underlying the metabolism of DAMP-adsorbing structures such as ROS-scavenging chemical units and cationic polymers remain unknown. While these structural motifs are common in materials, they are relatively new in the context of human medicine. With advancements in technology, such as nanomics, we now have the tools to assess the impact of material components on the body, although this field is still in the research and exploration stage [224]. Furthermore, DAMPs are not entirely harmful. Indiscriminate clearance of DAMPs may introduce new issues: Are there potential side effects? Could this lead to complications? Without a thorough understanding of the mechanisms and the materials’ mode of action, clinical translation is still a long way off.

### Biomaterials for cytokine neutralization

The straightforward method to reduce cytokines is the use of antibodies. However, antibodies are specific to certain cytokines and are expensive, with a certain degree of immunogenicity. In contrast, biomaterials capable of directly capturing and neutralizing cytokines have garnered substantial attention due to their low immunogenicity and multi-targeting capabilities [225]. Electrostatic binding with cytokines is a simple and effective mechanism. For example, glycosaminoglycan (GAG) hydrogels can adsorb cytokines due to the highly negatively charged sulfate and carboxyl groups in their molecular structure. These charges facilitate strong electrostatic interactions with the positively charged regions of cytokines, allowing GAG hydrogels to regulate the transmission of cytokines and influence their biological activity [226]. Additionally, the cell membranes wrapping NPs can inherit the antigenic outer surface and related membrane functions of the source cells [227]. The specific membrane proteins and lipids on these cell membranes such as selectins and integrins can actively bind to cytokines, enabling the NPs to capture and neutralize inflammatory molecules. By mimicking the natural surface of immune cells, these NPs can enhance the specificity and effectiveness of cytokine adsorption, providing a more versatile and targeted therapeutic approach.

Given the systemic inflammatory nature of RA, the second method is preferable. In 2018, a broad-spectrum anti-inflammatory strategy was developed for the treatment of RA based on neutrophil-like NPs [228]. Neutrophil membranes extracted from human peripheral blood were purified and coated onto PLGA polymer cores. The biomimetic NPs possess the surface antigen characteristics of neutrophils, enabling them to recognize and bind to inflammatory cytokines, thereby preventing the activation of endogenous neutrophils by cytokines. This innovative approach harnesses the natural capabilities of neutrophils to neutralize pro-inflammatory cytokines, offering substantial advancement in RA therapeutics. Unlike existing anti-cytokine drugs that target specific and limited objectives, this method achieves function-driven and broad-spectrum anti-inflammatory effects against multiple high-concentration inflammatory factors. In addition to neutrophil membranes, other immune cells such as monocytes, macrophages, DCs, and T cells can all be used as cell membrane coatings for targeted and synergistic therapy in RA.

A method was developed recently to obtain intracellular supramolecularly gelled macrophages (GMs) that effectively preserve intact cell membrane structures, including the content, variety, fluidity of membrane proteins, and the order of membrane lipids [229]. GMs have the potential to serve as multi-target anti-inflammatory adsorbents, which actively and efficiently adsorb and neutralize a diverse array of inflammatory cytokines, thereby enhancing the efficacy of multi-factor anti-inflammatory therapy. In a rat model of collagen-induced arthritis (CIA), GMs alleviated bone damage in joints and suppressed the overall symptoms of arthritis. This intracellular gelation technology, leveraging the antigenic structures on the membrane surface, offers a new broad platform for targeted inflammatory therapy in the treatment of RA.

GAG hydrogels and biomimetic NPs can adsorb and neutralize various cytokines through electrostatic interactions and cell membrane recognition. Compared to traditional antibody therapies, this approach has lower immunogenicity, reduced costs, and does not require specific design for targeting individual cytokines, making it highly promising for clinical applications. However, unlike DAMP adsorption, theoretical research on cytokine adsorption remains underdeveloped. Indiscriminate cytokine adsorption could also potentially neutralize cytokines that are beneficial to the body. Furthermore, the long-term safety, metabolic pathways, and the effects of cell membrane-coated NPs on the immune system are critical issues that cannot be overlooked before transitioning to clinical applications.

### Biomaterials for signal regulation

Although signaling pathways contribute to a crucial role in RA, direct modulation of these pathways using solely the structure of biomaterials remains challenging at present. The majority of studies focus on the relationship between signaling pathways and biofactors, aiming to indirectly regulate the biofactors or immune cells to investigate whether they can influence one or multiple signaling pathways. For example, a high-performance biomedical hydrogel, OD-PP@SeNPs, was developed with a triple cross-linking structure, injectability, self-healing, strong tissue adhesion, and biodegradability [230]. The triple cross-linking is achieved through dynamic Schiff base bonds, phenylboronate ester bonds, and hydrogen bonds among poly-l-lysine grafted with phenylboronic acid, oxidized dextran, and selenium NPs. This hydrogel exhibits ROS/pH responsiveness, allowing for the controlled release of SeNPs, which synergistically enhance antioxidant and anti-inflammatory ability. To further elucidate the mechanisms underlying their antioxidant and anti-inflammatory actions, the hydrogel was discovered to substantially decrease the levels of phosphorylated PI3K and phosphorylated AKT in the signaling pathways. Additionally, the hydrogel notably reduced the phosphorylation of ERK1/2, c-Jun N-terminal kinase (JNK), and p38. Concurrently, NF-κB signaling transduction was suppressed, and the expression of proteins in the NF-κB pathway was reduced by the hydrogel. Through these mechanisms, the hydrogel reshapes the immune microenvironment, effectively alleviating inflammation.

In addition, some methods utilize the sequence structure of biomaterials to achieve signal blocking. Programmable DNA origami technology was developed to regulate the CD95 death-inducing signaling in activated immune cells, establishing local immune tolerance in inflamed synovial tissues to reverse RA [231]. The signaling pathway mediated by CD95 and its ligand (CD95L) plays a pivotal role in the induction of immune tolerance toward self-antigens and the elimination of activated lymphocytes. The DNA origami technique facilitates the arrangement of a CD95L array in a 2-dimensional hexagonal pattern, with each molecule spaced approximately 10 nm apart, which corresponds to the geometric arrangement of clusters of transmembrane CD95 receptors. Furthermore, the DNA origami structures were equipped with I-motif DNA sequence-based fasteners, allowing for reversible switching between a closed and open configuration upon exposure to pH-induced stimuli. Therefore, the designed DNA origami maintains a closed conformation under neutral conditions and transits to an open conformation in a weakly acidic environment (pH ~6.5) to expose the special structure of the CD95L array, whereby the CD95 signals in activated immune cells can be selectively activated to precipitate their demise. This innovative material design enables spatial regulation of signaling pathways in immune cells and offers a novel perspective for the development of signal regulation therapies in the context of RA.

## Biomaterials for Endogenous Gas Regulation

Endogenous gas molecules such as H_2_ (hydrogen), H_2_S (hydrogen sulfide), O_2_ (oxygen), and NO play important roles in the immune microenvironment of RA and have a multifaceted impact on the disease’s treatment and pathophysiological processes. Some biomaterials are specifically engineered to precisely regulate the levels of these endogenous gas molecules, thereby modulating the immune microenvironment and promoting the repair of damaged tissues.

### Nitric oxygen clearance

NO is an important bioactive molecule, with its primary biological activity mediated through the nitrosylation of thiols, regulating various physiological and pathological processes such as apoptosis, vasodilation, and mitochondrial function [232,233]. In RA, elevated levels of NO and iNOS are positively correlated with disease progression, leading to exacerbated inflammation and cartilage destruction [234,235]. So far, numerous biomaterials have been designed to clear NO in situ at the joint to suppress RA damage caused by NO.

**Fig. 2.**
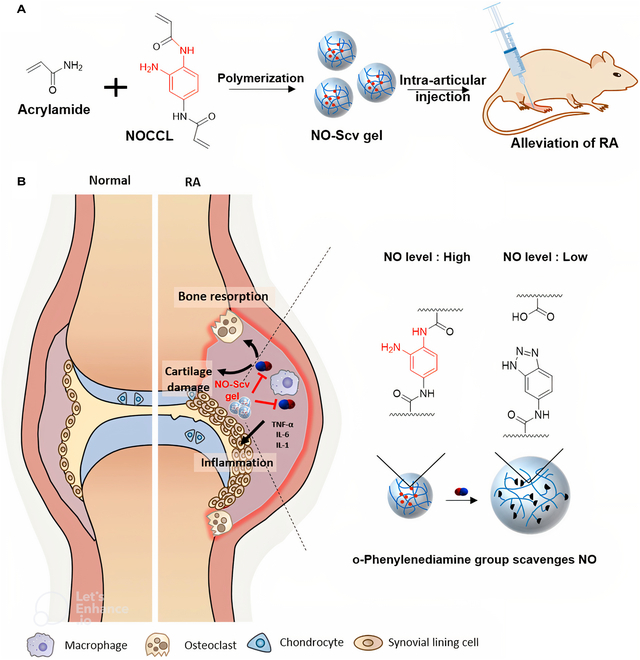
Schematic illustration of NO-Scv gel-mediated anti-inflammation therapy for treating RA. (A) Preparation and application of NO-Scv gel for treating RA. Intra-articular injection of NO-Scv gel into RA model mouse to alleviate RA by scavenging NO. (B) Mechanism of NO-induced inflammatory effect in RA and alleviation of RA by NO-Scv gel. NO directly damages cartilage, up-regulates osteoclasts inducing deformation of bone, and further aggravates the inflammation along with proinflammatory cytokines. Reprinted (adapted) with permission from [236]. Copyright (2019) American Chemical Society.

A novel NO-scavenging nanohydrogel, named NO-Scv gel [236], was prepared by controlling the concentrations of acrylamide and NO-cleavable crosslinking agents (NOCCL) through solution polymerization (Fig. 2). When injected into the joints of RA model mice, the nanohydrogel efficiently cleared excessive local NO, thereby inhibiting osteoclastogenesis and alleviating joint inflammation. To the best of our knowledge, this is the first report of a biomaterial specifically designed to mitigate RA by scavenging NO. This pioneering work establishes a new paradigm for the design of biomaterials aimed at modulating the local inflammatory environment in RA, potentially for increased safety and efficacy of treatment options for RA patients. In addition, there is a strategy that utilizes the inherent ROS- and NO-scavenging capabilities provided by DNA to decrease NO levels. The folate-modified triangular DNA origami nanostructures (FA-tDONs) actively target M1 macrophages in inflamed joints and promote the transition of macrophages from the M1 to M2 phenotype through ROS and NO clearance [237]. In a rodent RA model, the administration of FA-tDONs notably reduced synovial inflammation and cartilage degradation, thereby slowing disease progression.

### Hydrogen sulfide production

H_2_S is a small lipophilic molecule capable of penetrating cell membranes without requiring specific transport proteins, allowing it to exert effects on multiple targets within biological systems [238,239]. H_2_S possesses favorable biological effects in various aspects, including the cardiovascular system, immune cell regulation, cellular protection, anti-inflammation, and antioxidant activities [240–242]. In the context of RA, the most extensively studied function of H_2_S is its anti-inflammatory role [243]. H_2_S can suppress the expression of pro-inflammatory cytokines in various cells such as macrophages, synovial cells, T cells, and chondrocytes by inhibiting signal transduction pathways such as NF-κB and MAPK [244–246]. Additionally, under conditions of arthritis, H_2_S can induce bioactive factors for cartilage/bone repair, thereby ameliorating the bone destruction caused by RA [247,248]. Currently, exogenous small-molecule H_2_S donors such as ATB-346 and GYY4137 show marked potential in generating H_2_S to inhibit arthritis in vivo [249,250]. However, challenges remain in controlling the release rate of H_2_S and targeting specificity. Biomaterials that enable localized and sustained release of H_2_S offer a promising solution. A triblock copolymer with a core containing cysteine-triggered H_2_S donors [trithiocarbonate chain transfer agents (CTAs)] and NO-responsive N-(2-aminophenyl)acrylamide hydrochloride (NAPA) [251] achieves the release of H_2_S only in the presence of cysteine, preventing the rapid accumulation of H_2_S in biological systems.

### Oxygen generation

The synovial inflammatory response within the joints leads to the accumulation of a large number of inflammatory cells, which consume oxygen and result in local oxygen deficiency, thereby creating a hypoxic state in the joint microenvironment of RA [252]. Hypoxia induces an increase in hypoxia-inducible factor-1α (HIF-1α) and angiogenic factor VEGF, leading to alterations in cellular metabolic pathways, abnormal neovascularization, intensified inflammation, and joint damage [253]. Restoring oxygen pressure balance is crucial for alleviating RA symptoms. Clinically, hyperbaric oxygen therapy has been shown to reduce joint pain and stiffness associated with RA [254].

Currently, various studies are utilizing the structural properties of biomaterials to produce oxygen directly at inflammatory sites, thereby improving the symptoms of RA. Hydrogen peroxide (H_2_O_2_)-mimicking nanozymes, which can convert H_2_O_2_ into oxygen, offer a promising solution for diseases that require relief from oxidative stress and hypoxia [255]. Mesoporous manganese cobalt oxide (MnCoO) nanozymes with catalase (CAT)-like activity [256] were incorporated into a hydrogel matrix composed of polymeric hyaluronic acid to create a nanozyme-reinforced hydrogel [257]. This hydrogel effectively decomposes endogenous hydrogen peroxide to produce oxygen, alleviating hypoxia and oxidative stress in the RA microenvironment, while simultaneously promoting the proliferation and osteogenic differentiation of bone marrow stromal cells (BMSCs). A similar strategy was adopted to prepare hydrogen peroxide-mimicking enzymes using hydrated iron oxide NPs, which efficiently facilitate the localized conversion of hydrogen peroxide into oxygen, further alleviating oxidative stress and hypoxia within the RA microenvironment [258].

### Hydrogen release

H_2_ is an effective antioxidant and anti-inflammatory agent, capable of selectively neutralizing highly toxic reactive species such as ·OH and peroxynitrite anions (ONOO^-^). It also has the potential to enhance the activity of antioxidant enzymes such as CAT and superoxide dismutase (SOD), demonstrating promising potential in suppressing oxidative stress [259].

As previously mentioned, the progression of inflammation in RA is closely linked to oxidative stress, which is the consequence of the excessive production of ROS. Within the immune microenvironment of RA, hydrogen can inhibit oxidative stress and reduce inflammatory responses, positively impacting the pathological processes of RA [260]. However, the nonpolarity of hydrogen and its low solubility under physiological conditions limit its therapeutic efficacy. To address this, the design of biomaterials capable of generating hydrogen in situ at the inflammatory site to suppress oxidative stress in RA has been a major focus. For example, a biocompatible self-propelled magnesium–hyaluronic acid micromotor (Mg-HA motor) serves as an innovative approach for precise RA management [261]. These micromotors are engineered through an asymmetrical coating process, where the magnesium microparticles are first coated with a hyaluronic acid-loaded hydrogel, followed by a polymeric PLGA layer. Upon intra-articular injection, these micromotors generate hydrogen bubbles locally, which not only propel their movement but also act as active agents for scavenging ROS and inflammation. A novel yolk–shell heterostructure, H-AAZS (Au/Ag@ZnS-modified hyaluronic acid), is integrated with metal ions effectively [262]. By leveraging the unique structural properties of Au/Ag@ZnS, the separation and exploitation of photoinduced charge carriers have been improved, which in turn have promoted the sustained production of hydrogen when exposed to a 660-nm laser light. Both in vitro and in vivo experiments have demonstrated the material’s potential to mediate anti-inflammatory responses through hydrogen production in the treatment of RA.

### Carbon monoxide release

Carbon monoxide (CO) is another important endogenous gas. Due to its reducing ability, CO can scavenge ROS and participate in the regulation of the MAPK and JNK signaling pathways, inhibiting the release of pro-inflammatory cytokines such as TNF-α and IL-1β, making it a promising candidate for RA treatment [263]. Recently, ROS scavengers and CO-releasing agents have been integrated into a single platform, establishing a multifaceted management strategy to alleviate RA [264]. A novel molecular probe (named TTCO) consists of a long-wavelength-emitting aggregation-induced emission (AIE) core and a ROS-responsive manganese carbonyl cage. Upon encountering ROS, the manganese carbonyl cage undergoes cleavage, releasing the AIE luminophore (named TT), thereby remarkably enhancing the NIR-II fluorescence signal. Besides its imaging capabilities, TTCO also has the ROS-triggered therapeutic CO gas release. The simultaneous activation of imaging and therapeutic properties under inflamed ROS conditions makes the TTCO a promising candidate for precise RA diagnosis and on-demand treatment.

The use of chemical reactions to clear and release endogenous gases to improve the immune microenvironment is a remarkable advantage of biomaterials in the treatment of RA. This principle is similar to ROS scavenging, because it relies on the structural components of biomaterials to induce chemical reactions within the body, resulting in the reduction or production of specific substances that play a crucial role in RA treatment. However, the current strategies relying solely on endogenous gases for treatment remains insufficient. Without combining this approach with drugs, researchers and material scientists tend to focus more on designing novel, multifunctional molecular structures such as those with diagnostic luminescence or materials such as nanomotors rather than maximizing the therapeutic efficacy. Therefore, biomaterials that regulate the immune microenvironment through endogenous gases still have considerable development potential. Further research is needed to explore the role of endogenous gas molecules in RA and to understand how to maximize the therapeutic effects of these gas molecules. This will be key to advancing RA treatments and enhancing the efficacy of such biomaterials in clinical applications.

## Biomaterials for Cellular Modulation

The overactivated immune cells and excessive stimulation of resident tissue cells are primary drivers of RA pathology. These cells produce biological factors such as pro-inflammatory cytokines, inflammatory chemokines, and harmful substances, which further influence the activity, phenotype, and behavior of both themselves through signaling pathways. Currently, most biomaterial-based strategies to modulate cellular immune behaviors focus on indirectly regulating cells by modulating biological factors. Examples include regulating ROS to modulate macrophage phenotypes or controlling cytokine levels to influence T cell activity and phenotype. In this section, we will delve into the mechanisms by which biomaterials regulate immune cell behaviors and evaluate their potential for treating RA.

### Biomaterials for innate or tissue-resident cellular modulation

**Fig. 3.**
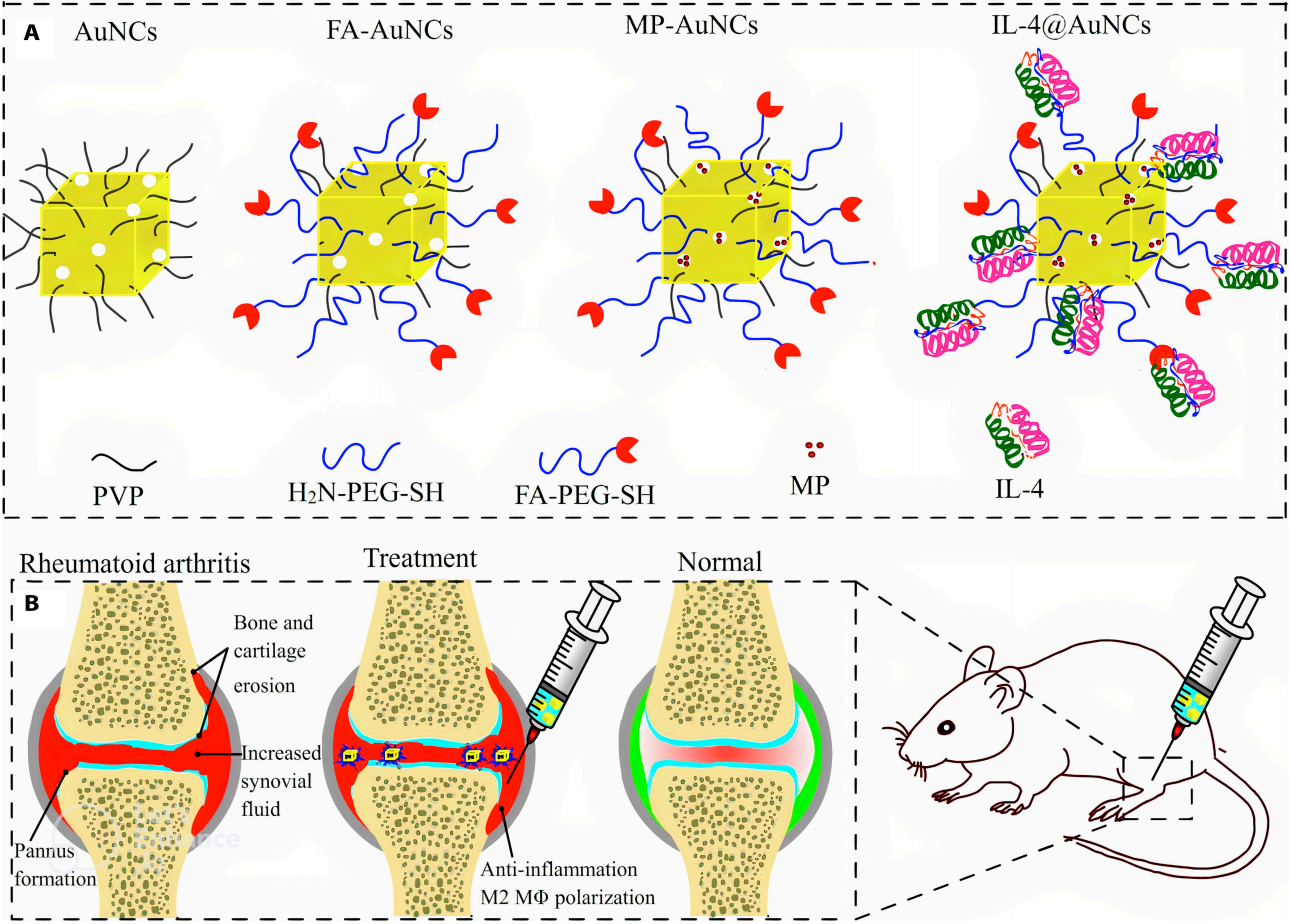
(A) Schematic representation of theranostic gold nanocage (AuNC) conjugates at different stages of fabrication [AuNCs: PVP-coated AuNCs; FA-AuNCs: folate acid (FA)-PEG-coated AuNCs; MP-AuNCs: FA-AuNCs loaded with methylprednisolone (MP); IL4-AuNCs: MP-AuNCs grafted with IL-4], and (B) intra-articular injection of the IL-4@AuNC targeting to folate receptor (FR) on the activated macrophages in RA joints, which release MP and recombinant IL-4 protein for the diagnosis and treatment. Reprinted from [269]. Copyright (2022) with permission from Elsevier.

Macrophage plasticity is a key characteristic, exhibiting heterogeneous phenotypes and various subpopulations. In RA, various factors, including pro-inflammatory agents such as inflammatory cytokines, chemokines, and DAMPs, drive macrophage polarization toward the M1 phenotype. The polarization rapidly triggers an “oxidative stress” response within minutes, resulting in the production of a large amount of pro-inflammatory substances that promote inflammatory responses [119,265]. Therefore, modulating inflammation to inhibit macrophage polarization has been a common approach. So far, most biomaterial strategies for regulating macrophages have been achieved by suppressing oxidative stress, because high levels of ROS can promote macrophage polarization toward the M1 phenotype by influencing macrophage metabolism and activating specific signaling pathways. For example, silver NPs (FA-AgNPs) can target M1 macrophages via folic acid (FA) receptors expressed on their surface and inhibit ROS production, thereby regulating macrophage phenotypes. Upon cellular internalization, the NPs dissolve and release Ag^+^ ions under the influence of intracellular glutathione (GSH). This process scavenges ROS, promoting the transition of macrophages from the pro-inflammatory M1 phenotype to the anti-inflammatory M2 phenotype [266]. Furthermore, anti-inflammatory cytokines, including IL-4 and IL-10, have been identified as instrumental in guiding macrophages toward an anti-inflammatory M2 phenotype [267,268]. Our team has engineered gold nanocages by adding HAuCl_4_ solution into Ag nanocubes and polyvinylpyrrolidone (PVP) mixed solution, capable of modulating macrophage polarization [269]. These nanocages loaded with methylprednisolone (MP) are functionalized with FA to target M1 and IL-4 to induce their transition to the M2 phenotype (Fig. 3). Both in vitro and in vivo studies have confirmed that these nanocages efficiently polarize macrophages toward the M2 phenotype without the need for MP and significantly reduce inflammation.

**Fig. 4.**
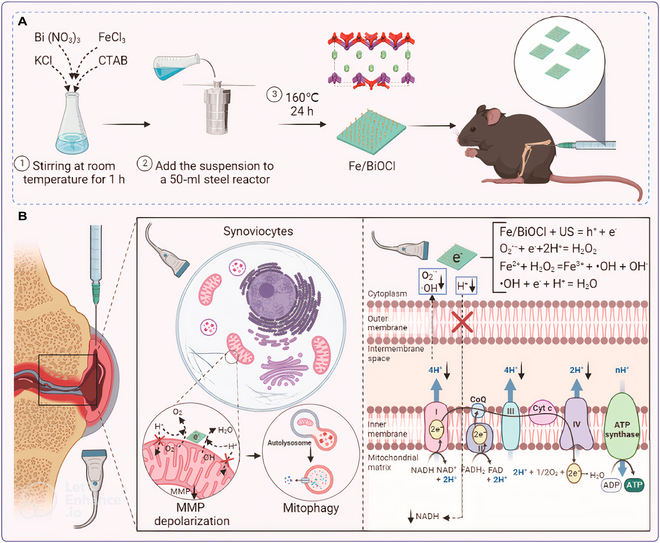
Schematic illustration of US-assisted Fe/BiOCl nanosheets (NSs) for RA. (A) The hydrothermal method was used to create Fe/BiOCl nanosheets. (B) After injection into the joint cavity, Fe/BiOCl penetrates synovial cells via cell-mediated entry and is targeted or adsorbed around the mitochondria due to its significant positive surface charge. Under US stimulation, the piezoelectric action of Fe/BiOCl produces electrons. The generated electrons can effectively bind to remove O_2_•^–^ and •OH produced by abnormal mitochondria while consuming a large amount of H^+^. Reprinted (adapted) with permission from [271]. Copyright (2024) American Chemical Society.

Excessive ROS primarily originate from mitochondrial dysfunction within cells [270]. In addition to directly scavenging ROS, an alternative strategy involves modulating ROS by restoring mitochondrial abnormalities, thereby inducing macrophage polarization toward the M2 phenotype [271]. An iron-doped piezoelectric material Fe/BiOCl, with a positive charge on its surface provided by cetyltrimethylammonium bromide (CTAB), can target the mitochondria of RA-FLSs and generate electrons under ultrasound (US) to remove O_2_•^−^ and •OH from the ROS-rich environment (Fig. 4) [271]. This process consumes hydrogen ions (H^+^), disrupting the internal hydrogen ion supply within the mitochondrial matrix. The resulting disruption triggers mitophagy and reduces the production of inflammatory mediators such as ROS, leading to macrophage repolarization and inflammation prevention.

Regulation of ROS also influences osteoclasts [272]. The lentinan-Se (LNT-Se) NPs can rapidly metabolize in vivo to selenocysteine, the predominant active form of selenium, which up-regulates the expression of glutathione peroxidases (GPx) to alleviate cellular damage caused by excessive ROS [273]. Specifically, the LNT-Se NPs enhance the levels of selenoenzymes, counteracting the pathological conditions associated with heightened oxidative stress during osteoclastogenesis. This intervention regulates the M1/M2 macrophage balance and diminishes osteoclastogenesis in vitro and in the CIA model in vivo. Osteoblast regulation is equally indispensable for restoring bone balance in RA. For example, NPs with a Zn-curcumin (Zn-Cur) core formed through chelate interaction between curcumin and Zn^2+^ and a hybrid framework of copper silicate as a shell [274] offer a dual-function approach. The copper silicate nanocarriers can degrade in the acidic microenvironment of the arthritic region, releasing Cu^2+^ and Zn-Cur. Subsequently, copper ions replace zinc ions, while the released Zn^2+^ and silicate synergistically promote biomineralization of BMSCs, thereby facilitating bone regeneration.

**Fig. 5.**
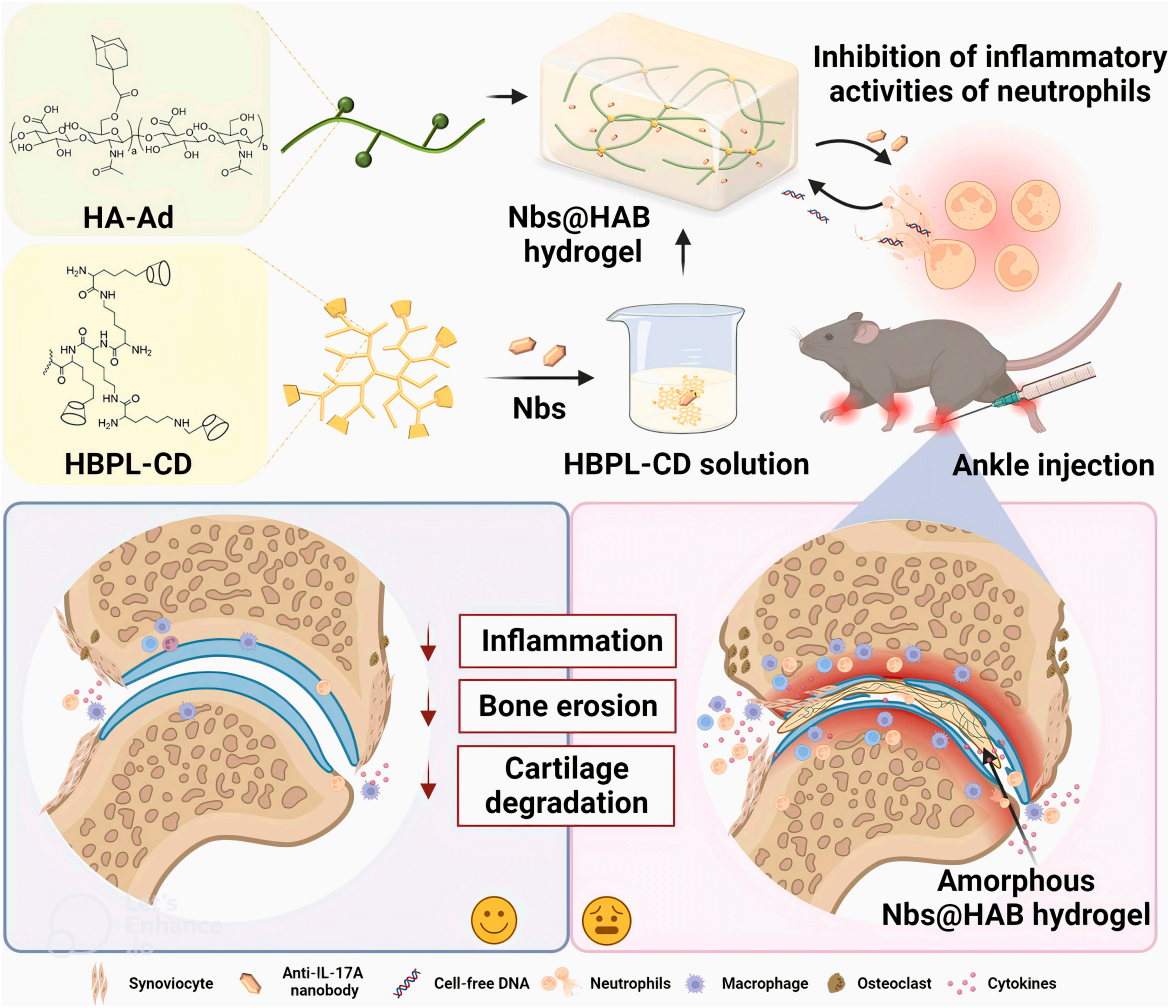
Schematic illustration of Nbs@HAB hydrogel for RA treatment via inhibition of inflammatory activities of neutrophils. Nbs are added to the HBPL-CD solution before gelation. The hyperbranched polylysine modified by β-cyclodextrin (HBPL-CD) and adamantane-modified hyaluronic acid (HA-Ad) form the hydrogel by host–guest interaction. After ankle injection, the Nbs@HAB hydrogel resides in the joint cavity, with the ability of adsorbing cell-free DNA (cfDNA) persistently and slowly releasing anti-IL-17A Nbs, which synergistically alleviates the inflammatory activities of neutrophils and suppresses the RA symptoms. Reprinted from [277]. Copyright (2024) with permission from Elsevier.

Although neutrophils are a crucial component of innate immunity, their modulation using biomaterials remains relatively rare. This scarcity is partly due to the challenges associated with neutrophil research and the current depth of understanding within the field [103]. Although the neutrophils cannot be definitely categorized into phenotypes 1 and 2 that can interconvert under different conditions, the existence of N1 and N2 neutrophil phenotypes in cancer suggests the potential for neutrophil differentiation into distinct phenotypes [275,276]. Moreover, the unique pathological features of neutrophils in RA, such as NETs, provide an ideal target for neutrophil regulation. For instance, we have developed a cationic polymer hydrogel (HAB hydrogel) loaded with nanobodies (Nbs) that can mitigate the harmful effects of NETs in the RA immune microenvironment. The hydrogel’s cationic polymer component, hyperbranched polylysine, binds to NET-derived DNA through electrostatic interactions, thereby reducing its damaging effects. Additionally, we have investigated the potential of this hydrogel to regulate neutrophil phenotypes and reduce the secretion of neutrophil inflammatory factors, providing a new direction for the regulation of neutrophils in the treatment of RA (Fig. 5) [277].

### Biomaterials for adaptive cellular modulation

Adaptive immunity is crucial to the clinical symptoms of RA. Suppressing the overactive adaptive immune response in RA remains a major challenge for pharmacotherapy and biomaterials research. Mainstream studies have focused on the “pre-RA” stage, targeting adaptive immune cells such as DCs or T cells that can differentiate into immunosuppressive cells in vivo [278]. By loading autoantigens or immunomodulatory small molecules, various tolerance-inducing NPs (or immunomodulatory nanopreparations) have been developed to reshape the immune response and ultimately induce antigen-specific immune tolerance before the onset of RA, thereby inhibiting the development of RA [64]. For instance, systemic delivery of NPs coated with autoimmune disease-relevant peptides bound to MHC-II molecules (pMHC-II) could trigger and expand antigen-specific regulatory CD4^+^ T cell type 1 (T^+^_R_1)-like cells [279]. These NPs can resolve established autoimmune phenomena in different mouse models, such as mice humanized with patient-derived lymphocytes. However, this strategy primarily represents another form of drug delivery, and the heterogeneity among RA patients means that different individuals may require different autoantigens, greatly limiting the broad application of such methods. Moving beyond traditional approaches that rely on the delivery of self-antigens or immunomodulatory small molecules, an innovative nanohybrid therapy, the ceria-vesicle nanohybrid therapeutic, has been developed [280]. The therapy involves immobilizing cerium oxide NPs onto mesenchymal stem cell-derived nanovesicles, creating a hybrid system capable of coordinating both innate and adaptive immunity. The cerium NPs (Ce NPs), acting as antioxidants, scavenge ROS and induce macrophage polarization from the M1 to M2 phenotypes. Meanwhile, the mesenchymal stem cell nanovesicles (MSCNVs) protect chondrocytes, prevent bone and cartilage damage, and deliver immunomodulatory cytokines to target cells like DCs and T cells, guiding them toward an immunoregulatory phenotype. This approach bridges the gap between the modulation of innate and adaptive immune responses through both individual and combined effects, aiming to rapidly alleviate inflammatory symptoms and restore the immune system function of RA.

Immunity lies at the core of RA, and cellular behavior is the key to achieving immune regulation. It is evident that current biomaterial strategies for cell regulation in RA treatment are far fewer and less developed compared to the well-established FDA-approved antibody therapies. Most strategies primarily regulate cellular behaviors indirectly by modulating biological factors. In many cases, research combines biomaterials with delivery agents such as drugs, antibodies, or exosomes to achieve synergistic and more effective therapeutic outcomes.

Nonetheless, with a deeper understanding of RA pathology and the growing recognition of the importance of immune cells, the focus of research in this field is gradually shifting. It is expanding from macrophage regulation to strategies that include neutrophils and osteoclasts, and even multifactorial approaches targeting both innate and adaptive immunity. The future of leveraging biomaterials for efficient cell regulation in RA treatment is promising and not far off.

## Conclusions and Future Perspectives

The emergence of biomaterials capable of modulating the immune microenvironment has marked a notable shift in the treatment paradigm for RA. These innovative materials offer a comprehensive strategy for addressing the complex immune dysregulations underlying RA. Moreover, the understanding of microenvironment in RA is crucial for guiding the concurrent development of biomaterials to intervene in this multifaceted pathological process.

Biomaterials harness their chemical and physical properties to perform critical functions such as adsorbing or neutralizing cytokines and harmful DAMPs through electrostatic interactions, clearing local harmful substances, and generating beneficial gases by structural degradation or chemical bond breakage. Their material forms and modifiability allow for precise targeting of immune cells and local immune microenvironment, enabling controlled therapeutic interventions in both time and space. By leveraging their structural advantages, biomaterials primarily address 3 critical aspects: regulating biofactors and signaling pathways, controlling endogenous gas, and modulating key cellular functions. Through these regulatory mechanisms, biomaterials have paved the way for innovative management strategies in this debilitating autoimmune disease, offering a new frontier in RA treatment.

With a deepened understanding of the immunological microenvironment in RA, biomaterials have evolved from passive drug carriers to active immunomodulators. The unique advantages of biomaterials, such as their customizability, processability, biocompatibility, morphological flexibility, shorter production cycles, and lower development costs, position them as a novel therapeutic approach independent of traditional drugs, actively contributing to the advancement in RA prevention and therapy. Although the independent use of biomaterials to modulate the immune microenvironment is still in its infancy compared to the drug delivery systems, existing research has provided new perspectives and directions for RA treatment. Researchers can draw upon interdisciplinary knowledge from physics, chemistry, biology, and material science to optimize the composition, form, and structure of biomaterials. Novel biomaterials not only replicate the functions of drugs, such as broad-spectrum anti-inflammatory effects, antioxidant capabilities, modulation of innate and adaptive immune responses, and blockade of pro-inflammatory cytokine signaling, but, in some cases, even surpass the efficacy of pharmaceuticals. Smart materials, biomimetic materials, and adaptive materials are being developed to specifically target the immunological microenvironment of RA, making biomaterials an independent therapeutic approach that offers new therapeutic insights and directions.

However, challenges remain in the development of novel biomaterials for RA therapy. The precise control of biomaterial properties such as structure, composition, size, and shape requires further investigation to optimize their therapeutic potential. Additionally, issues related to toxicity and metabolic interactions that may arise during the adsorption or interaction of biomaterials with the microenvironment need to be addressed. The successful translation of functional biomaterials from the laboratory to clinical applications is a common challenge faced by all functional biomaterials. Currently, the range of targets and design methods for modulating the RA immunological microenvironment is still relatively limited. Looking ahead, with the continuous advancement of biology, medicine, chemistry, and materials science, alongside the integration of interdisciplinary approaches, it is anticipated that more innovative regulatory methods and novel biomaterial strategies will emerge, providing new impetus and solutions for RA treatment.

## Data Availability

No new data were created or analyzed in this study.
